# Transcriptomics Reveals an Energy-Saving Metabolic Switch in an Extremophilic Red Microalga *Cyanidioschyzon merolae* Under Nickel Stress

**DOI:** 10.3390/ijms26104813

**Published:** 2025-05-17

**Authors:** Sergio Santaeufemia, Francesca Marchetto, Patrizia Romano, Dorota Adamska, Krzysztof Goryca, Jeffrey Palatini, Joanna Kargul

**Affiliations:** 1 Solar Fuels Laboratory, Center of New Technologies, University of Warsaw, 02-097 Warsaw, Poland; sergio.santaeufemia-sanchez@tum.de (S.S.); f.marchetto@cent.uw.edu.pl (F.M.); 2 Department of Physical Sciences, Earth and Environment, University of Siena, 53100 Siena, Italy; patrizia.romano@student.unisi.it; 3 Genomics Core Facility, Centre of New Technologies, University of Warsaw, 02-097 Warsaw, Poland; d.adamska@cent.uw.edu.pl (D.A.); k.goryca@cent.uw.edu.pl (K.G.); j.palatini@cent.uw.edu.pl (J.P.)

**Keywords:** *Cyanidioschyzon merolae*, heavy metals, nickel, photosynthesis, adaptation, transcriptomics

## Abstract

The red microalga *Cyanidioschyzon merolae* inhabits extreme environments with high temperatures (40–56 °C), high acidity (pH 0.05–4), and high concentrations of heavy metals that are lethal to most forms of life. However, information is scarce on the precise adaptation mechanisms of this extremophile to such hostile conditions. Gaining such knowledge is important for understanding the evolution of microorganisms in the early stages of life on Earth characterized by such extreme environments. Through an analysis of the re-programming of the global transcriptome upon the long-term (up to 15 days) exposure of *C. merolae* to extremely high concentrations of nickel (1 and 3 mM), the key adaptive metabolic pathways and associated molecular components were identified. Our work shows that the long-term Ni exposure of *C. merolae* leads to the lagged metabolic switch demonstrated via the transcriptional upregulation of the metabolic pathways critical for cell survival. DNA replication, cell cycle, and protein quality control processes were upregulated, while downregulation occurred with energetically costly processes, including the assembly of the photosynthetic apparatus and lipid biosynthesis. This study paves the way for future multi-omic studies of the molecular mechanisms of abiotic stress adaptation in phototrophs, as well as the future development of rational approaches to the bioremediation of contaminated aquatic environments.

## 1. Introduction

The rapid advancement of RNA sequencing technologies enables in-depth insight into the molecular mechanisms of adaptation to various abiotic and biotic stressors such as exposure to heavy metals. As an example, the transcriptomic studies on the effects of Cd or Ag in a green microalga *Chlamydomonas reinhardtii* showed that genes belonging to metabolic pathways of oxidative stress response and photosynthesis were regulated via these metals [1,2]. In a diatom *Phaeodactylum tricornutum*, the analysis of the transcriptome showed that Ni-regulated genes involved in metabolic pathways related to the nitrogen cycle, DNA structure remodeling and replication, fatty acids biosynthesis, and the regulation of the thiol-disulphide redox system [3].

Nickel is a toxic heavy metal for both eukaryotic and prokaryotic organisms, inducing the generation of reactive oxygen species (ROS), which, in turn, can cause cellular damage through reactions with proteins and DNA.

In particular, the Ni affinity for the -SH groups of enzymes and other proteins and the competition of Ni with other metal cations cause the depletion of enzyme activity, which negatively affects various cellular processes, including cell division, respiration, and photosynthesis [4]. Although the high toxicity of Ni and the detrimental effects of this heavy metal on cellular metabolism is well-documented, knowledge of transcriptome changes caused by Ni exposure is limited, especially in relation to extremophilic microalgae that are naturally exposed to high concentrations of heavy metals. These include members of the Cyanidiales order of thermo-acidophilic red microalgae inhabiting aquatic environments of volcanic origin. A representative of this fascinating group of extremophilic microorganisms, *Cyanidioschyzon merolae*, was isolated from the volcanic hot springs of Campi Flegrei, Italy [5], and was shown to thrive in moderately high temperatures (up to 57 °C), with an extremely acidic pH (0.05–3), and in the presence of high levels of various heavy metals such as Co, Cd, Hg, Ni, As, Pb, Cr, and Fe [6]. Due to its unique evolutionary position at the root of the red algal lineage, this microalga represents a link between prokaryotic cyanobacteria and photosynthetic eukaryotes demonstrated, for example, through the hybrid characteristics of the photosynthetic apparatus [7,8]. The complete sequencing of its nuclear, mitochondrial, and chloroplast genomes [9] has established *C. merolae* as a model organism for a large number of evolutionary studies and for the dissection of the molecular mechanisms of various fundamental cellular processes, including circadian rhythms, lipid, and protein homeostasis, cell cycle and division [10,11,12,13], and, more recently, oxygenic photosynthesis [8,14,15,16,17,18,19,20].

In this study, we explored how long-term exposure to high nickel concentrations affected the transcriptional regulation of metabolic pathways in *C. merolae*. We focused on the identification of time-dependent changes in gene expression, particularly those involving pathways essential for cell survival—such as DNA replication, cell cycle progression, and protein quality control—and how these may be balanced via the downregulation of energetically demanding processes such as photosystems’ assembly.

## 2. Results and Discussion

### 2.1. Dynamic Remodeling of C. merolae Transcriptome During Long-Term Ni Adaptation

**Figure 1:**
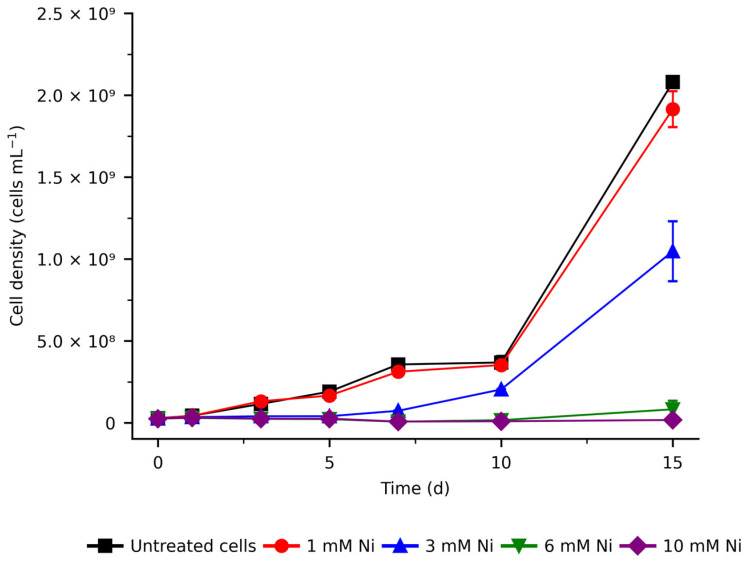
Growth curves of *C. merolae* cells exposed to various Ni concentrations. Cell density average values (±SD) were determined as described in Materials and Methods for up to 15 days of the Ni exposure for three independent biological replicas (*n* = 3).

In this study, we examined the re-programming of the global transcriptome of *C. merolae* during the long-term adaptation to extremely high concentrations of Ni. The Ni concentrations used (1–10 mM Ni) are up to seven orders of magnitude higher than those found in the natural habitat of this extremophilic microalga (0.003–0.155 mM Ni, [21]). Figure 1 shows the growth dynamics of *C. merolae* under exposure to 1–10 mM Ni for up to 15 days. As previously shown in Marchetto et al. (2024) [22], the cultures treated with 1 mM Ni showed no significant difference in cell growth compared to untreated control, reaching cell density values of, respectively, 2.08 × 10^9^ and 1.92 × 10^9^ cells mL^−1^ on day 15. The cell growth was significantly inhibited at 3 and 6 mM Ni from 24 h onward, reaching cell density values of 0.08 × 10^9^ and 0.02 × 10^9^ cells mL^−1^, respectively, on day 15. In contrast, cells exposed to 3 mM Ni initially showed growth inhibition during the lag phase (up to day 7), followed by a recovery phase between days 10 and 15, reaching 1.05 × 10^9^ cells mL^−1^ on day 15 (Figure 1).

To gain insight into metabolic remodeling during Ni adaptation, we conducted an in-depth analysis of the global transcriptome in response to Ni levels that yielded viable cells, i.e., up to 3 mM Ni. This Ni concentration is lethal for most other organisms, including the model mesophilic green microalga *Chlamydomonas reinhardtii* [23]. We analyzed 4765 genes at three different timepoints (days 5, 10 and 15) during 1 and 3 mM Ni exposure. Compared to the untreated control, we identified 252 and 2888 significantly differentially expressed genes (DEGs; adjusted *p*-value < 0.05; fold change (FC) > 1 for upregulated or FC between 0 and 1 for downregulated genes) for *C. merolae* cells exposed to 1 and 3 mM Ni, respectively, compared to the untreated control.

**Figure 2:**
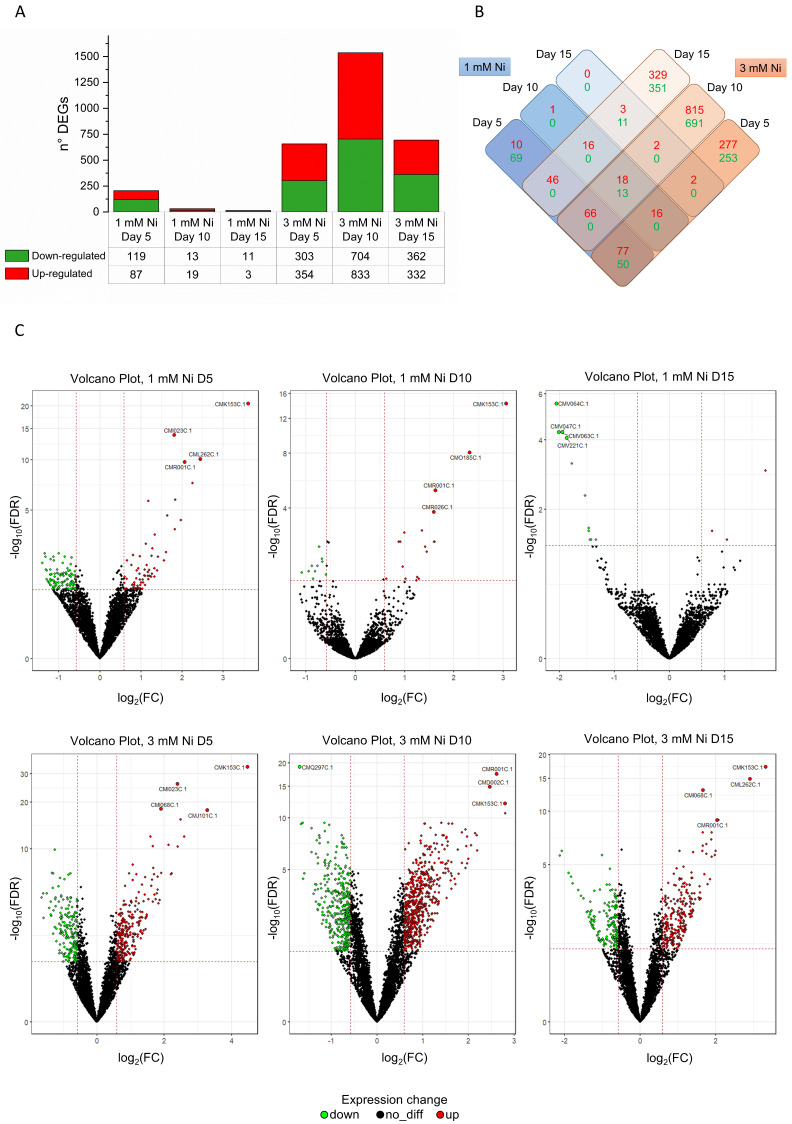
Differentially expressed genes in *C. merolae* cells exposed to 1 and 3 mM Ni. (**A**) Numbers of up- and downregulated genes for the different Ni concentrations and timepoints (5, 10, and 15 days). (**B**) Matrix of DEGs that were either unique or shared between Ni concentrations tested. Color coding: red, upregulated genes; green, downregulated genes. (**C**) Volcano plots of DEGs for untreated *C. merolae* cells versus cells exposed to 1 and 3 mM Ni at different timepoints. Plotted are log 10 (FDR—false discovery rate) versus log 2 (FC). Data were obtained from three biological replicas (*n* = 3).

The large number of DEGs (Figure 2A–C) suggests the presence of tightly coordinated metabolic responses during the Ni adaptation of *C. merolae*. The over 10-fold higher number of DEGs at 3 mM Ni compared to the 1 mM indicates substantial transcriptomic remodeling in this extremophile, which correlates with the cell growth dynamics observed for 3 mM Ni (Figure 1). Figure 2A–C shows that the cells treated with 1 mM Ni exhibit the highest number of DEGs on day 5, followed by the gradual decline. Interestingly, in the case of 3 mM Ni adaptation, the highest number of DEGs was observed on day 10, which corresponds to the onset of the recovery phase of cell growth (Figure 1).

**Figure 3:**
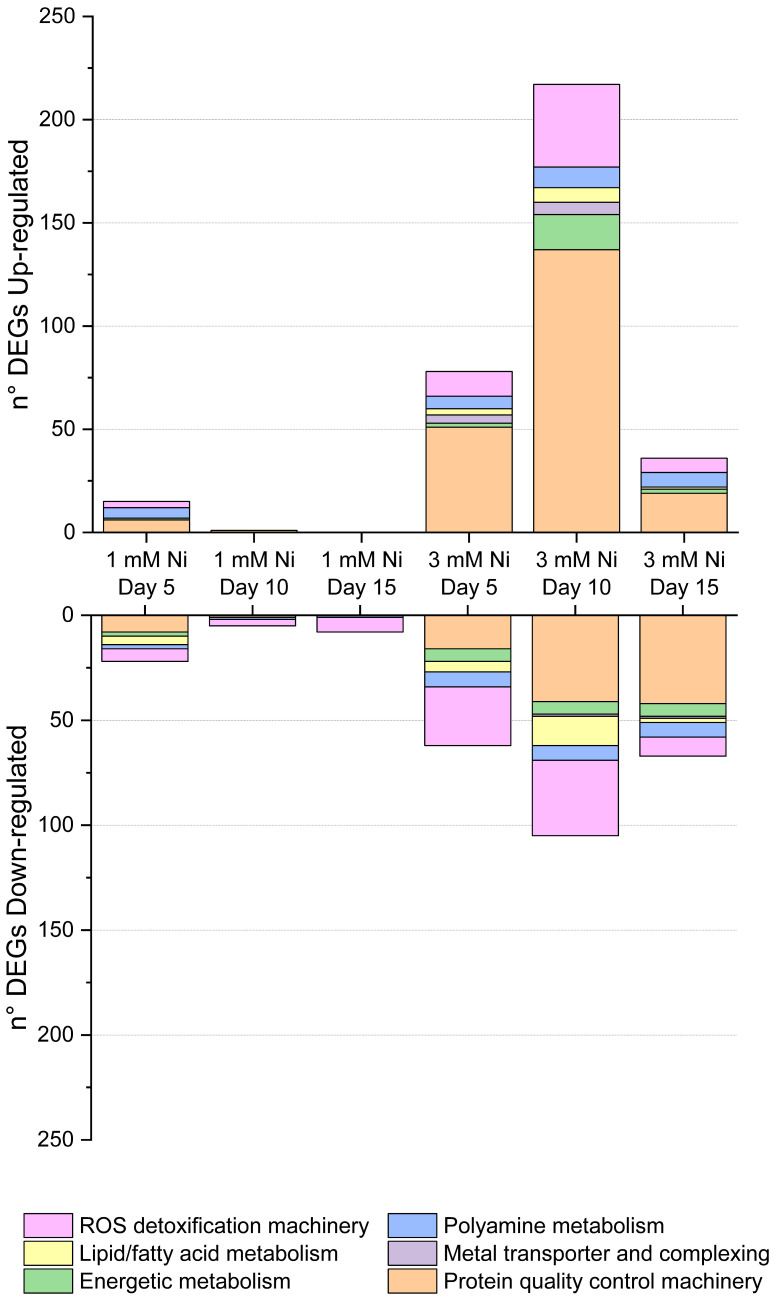
Dynamic regulation of differentially expressed genes associated with the specific cellular function during *C. merolae* exposure to 1 and 3 mM Ni. Up- and downregulated DEGs are grouped based on their metabolic function, including ROS detoxification pathways, lipid metabolism, energy metabolism, polyamine metabolism, metal transport and complexing, protein quality control, and DNA repair.

The DEGs shown in Figure 3 and listed in Table S1 reflect the intricate, dynamic remodeling of the transcriptome during the long-term exposure of *C. merolae* cells to Ni. Differentially regulated DEGs associated with the same metabolic function were observed at different timepoints. Below, we present a comprehensive analysis of transcriptomic changes associated with DNA replication and structural remodeling, cytoskeleton remodeling, oxidative stress responses, intra/extracellular metal transport and sequestration, and protein quality control, as well as lipid and energy metabolism.

### 2.2. DNA Replication and Structure Remodeling During Ni Adaptation

**Figure 4:**
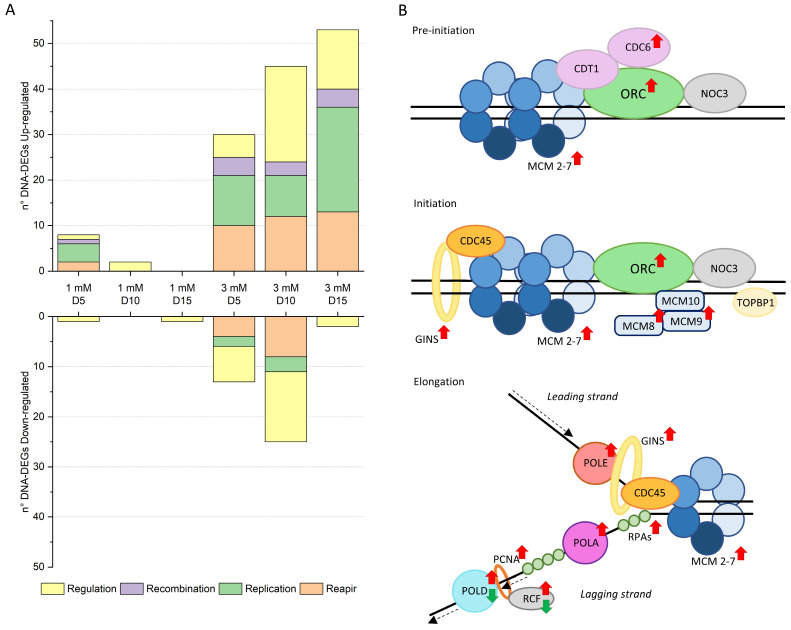
Ni effect on transcriptomic regulation of DNA metabolism. (**A**) DNA-related up- and downregulated DEGs classified by function. The DEGs related to different DNA function are divided into four categories: Repair, Replication, Recombination, and Regulation. The Repair and Replication categories include all entries that have a DNA repair or replication function, respectively. The Recombination category comprises DEGs that play a role in the recombination at the DNA or chromosomal levels, while the Regulation category clusters all entries whose function is associated with the regulation of various cellular processes, such as the cell cycle, chromosome segregation, histone modification, and DNA repair and replication. For the DEGs that have multiple functions (e.g., helicase), the entry is shown in all the relevant categories. The figure is based on data reported in Table S2. (**B**) Ni transcriptomic regulation of DNA replication machinery from pre-initiation to elongation phases. Red and green arrows represent up- and downregulated DEGs, respectively. For a description of the Ni treatment and timepoints, see Table S1. Adapted from Shultz et al. (2007) [24].

Long-term Ni exposure triggered dynamic transcriptomic remodeling in *C. merolae*, particularly in DNA-related pathways; 1 mM Ni-treated cells exhibited the highest number of DEGs on day 5, while 3 mM Ni-exposed cells peaked at day 10 indicating treatment-specific temporal response (Table S2). Specifically, the highest expression of genes involved in cell cycle regulation, chromosome segregation, histone modification, and DNA repair was observed on day 10, while a shift towards the upregulation of DNA replication-related genes was apparent on day 15 (Figure 4A). These findings highlight a time-dependent shift in the critical cellular responses during Ni adaptation.

Heavy metals including Ni are well documented as impairing DNA integrity by inhibiting synthesis, increasing mutation rates, and inducing chromosomal aberrations [4,25]. Cells counteract these effects by activating DNA repair and replication molecular machinery, regulated via chromosome segregation, as well as epigenetic modifications.

During the 1 and 3 mM Ni treatments, transcripts linked to the replication pre-initiation complex (pre-RC) were upregulated (Figure 4B, Tables S1 and S2), including MCM1 (*CMD025C*), MCM4 (*CMP365C*), MCM5 (*CMF173C*), MCM6 (*CMJ261C*), and MCM7 (*CMR234C*), indicating enhanced replication licensing during the early G1 phase [26]. The selective upregulation of additional components crucial to DNA synthesis [24], like MCM8 (*CMT087C*), MCM9 (*CMO137C*), and the entire GINS complex (Psf1 *CMR136C*, Psf2 *CMQ381C*, Psf3 *CMP212C*, and Sld5 *CMR406C*) suggests a targeted enhancement in replication efficiency under stress in *C. merolae*.

In addition to the preemptive replication machinery upregulation, we also observed a coordinated upregulation of key components of the DNA replication machinery: POLA (*CMF127C*, *CMI176C*), POLD (*CMB052C*, *CMN199C*), and POLE (*CMH082C*), as well as RFC2 (*CMF110C*) and PCNA (*CMS101C*), (Figure 4B). These proteins function in both replication and repair, reinforcing genome stability under Ni stress. The upregulation of repair genes, including MRE11 (*CMB035C*), MSH4 (*CMK199C*, *CMB035C*) MSH6 (*CMI229C*), and TFIH family members (*CMT290C*, *CME176C*), further confirms the activation of damage-control mechanisms [27]. Such patterns mirror mammalian systems, where increased replication activates MSH/MLH genes [28]. The upregulation of both replication and repair machinery represents a strategic cellular response in *C. merolae* to preserve genomic integrity in a challenging environment as under Ni-induced stress.

Another DEG cluster upregulated at 3 mM Ni (days 10 and 15) involved SMC (structural maintenance of chromosome) complexes, which support chromosomal transmission, condensation, and segregation, and DNA repair and recombination, as well as epigenetic silencing [29,30]. Notably, genes encoding the cohesin subunits SMC1-SMC3 (*CMI192C-CML027C*) and Dcc 1 (*CMN029C*) were upregulated, as were condensin components SMC2-SMC4 (*CMG189C-CME029C*), subunit G (*CMS422C*) and condensin II subunits D3 (*CMQ236C*), G2 (*CMA089C*), and H2 (*CMI207C*). These findings support the presence of active regulation of chromosomal architecture in *C. merolae* cells during Ni adaptation.

Additionally, SMC5-SMC6 (*CMH246C-CMA066C*), involved in the ds break DNA repair [31], were also upregulated. Genes associated with the kinetochore and spindle assembly checkpoint (SAC) were induced (Tables S1 and S2), all four Ndc80 complex subunits (*CMG078C*, *CMB012C*, *CMG186C*, *CMI101C*), Bub1/Mad3 (*CMK144C*), Mps1 (*CMB064C*, *CMB108C*), and SKA1 (*CMI099C*), indicating tight control of mitotic chromosome alignment [32].

Among the downregulated DEGs, most clustered around histone acetylation and methylation or methyltransferase activity (Table S2), suggesting reduced in chromatin accessibility and a possible transcriptional silencing response in *C. merolae* cells under Ni stress.

Moreover, SAC-related and microtubule-associated genes were consistently upregulated at 3 mM Ni on day 10, indicating reinforcement of the cytoskeletal integrity during cell division.

Together, these transcriptomic responses—the upregulation of repair, replication, and chromatin organization genes, alongside suppression of histone modifications—suggest a tightly controlled adaptation mechanism driving cell recovery from day 10 onward during 3 mM Ni exposure of *C. merolae* cells (Figure 1, Tables S1 and S2).

### 2.3. Regulation of Oxidative Stress Responses

**Figure 5:**
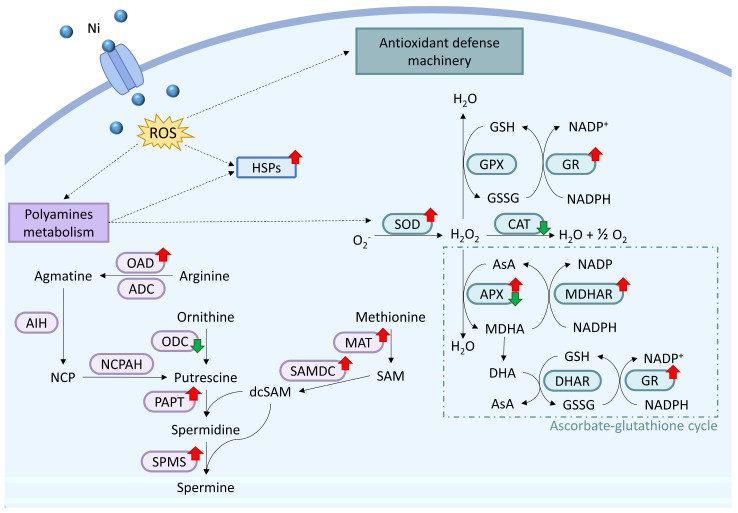
Ni-oxidative response machinery in *C. merolae*: antioxidant enzymatic defense and polyamine metabolism. Nickel, upon entering *C. merolae* cells, causes an increase in ROS levels, which upregulates the enzymatic antioxidant system, polyamine (PA) metabolisms, and heat-shock proteins (HSPs). PAs, in turn, upregulate HSPs and superoxide dismutase (SOD). For a description of timepoints and displayed enzymes, see Table S1. The dotted arrow indicates an upregulation of the metabolic pathway and specific molecular components. The red and green arrows correspond to up- or down-regulation of specific components, respectively. OAD, ornithine/arginine decarboxylase; ADC, arginine decarboxylase; AIH, agmatine iminohydrolase; NCPAH, N-carbamoyl putrescine amidohydrolase; ODC, ornithine decarboxylase; PAPT, putrescine aminopropyl transferase; SPMS, spermine synthase; MAT, methionine adenosyl transferase; SAMDC, S-adenosylmethionine decarboxylase; GPX, glutathione peroxidase, GR, glutathione reductase; CAT, catalase; APX, aspartate peroxidase; MDHAR, monodehydroascorbate reductase; DHAR, dehydroascorbate reductase; NCP, N-carbamoyl putrescine; dcSAM, decarboxylated S-adenosylmethionine; SAM, S-adenosyl methionine; GSH, glutathione; GSSG, glutathione disulfide; Asa, ascorbate; MDHA, monodehydroascorbate; DHA, dehydroascorbate.

Our recent study demonstrated that *C. merolae* cells exposed to 1–10 mM Ni for 15 days display a dynamic amelioration of the ROS accumulation between days 10 and 15, especially for 3 mM Ni-exposed cells [22]. The extreme environment in which *C. merolae* thrives is rich in heavy metals, which can induce oxidative stress, the indirect formation of ROS, and ROS-induced lipid peroxidation [4]. High ROS levels can ultimately damage membranes and enzymes and induce apoptosis. The dynamic growth of *C. merolae* under Ni suggests that this alga has evolved efficient responses to oxidative stress. Indeed, the DEG analysis revealed key components involved in ROS detoxification during Ni adaptation (Figure 5, Table S1).

Mn superoxide dismutase (MnSOD) was downregulated in a non-specifically localized variant on day 10 of 3 mM Ni adaptation, whereas the mitochondrial counterpart was upregulated both on days 5 and 10 (Figure 5 and Table S1). This suggests that the transcriptional activation of mitochondrial MnSOD is the primary response to increased ROS levels. However, other ROS-processing enzymes, like catalase (CAT) and glutathione peroxidase (GPX), were either downregulated or unchanged compared to the control (Figure 5, Table S1), highlighting a fine-tuned oxidative stress regulation involving various components of the ROS detoxification pathways.

Our findings highlight the importance of the ascorbate–glutathione cycle (AsA-GSH) in Ni-induced oxidative stress response. The upregulation of cytosolic ascorbate hydrogen peroxidase (APX), monodehydroascorbate reductase (MDHAR), and glutathione reductase (GR) suggests that this pathway plays a major role in *C. merolae*’s Ni adaptation (Figure 5). This is consistent with a similar observation in higher plants, where metal stress upregulates the same pathway [33]. Interestingly, genes involved in ROS scavenging, such as glutathione (GSH) and phytochelatins (PCs), remain unchanged, although two genes encoding glutathione S-transferase (GST) were upregulated, pointing towards their role in Ni-induced oxidative stress response in *C. merolae*.

During the recovery phase of Ni adaptation, biotin biosynthesis was upregulated (Table S1), consistent with findings in *C. reinhardtii* under mercury stress [34], indicating an evolutionary conserved adaptive response to heavy metal-induced oxidative stress. We also observed an intriguing upregulation of mercuric reductase, an enzyme that catalyzes the bio-transformation of Hg^2+^ into Hg^0^ and metacinnabar (β-HgS). This enzyme, considering also its activity in another extremophilic red microalga closely related to *C. merolae*, *G. sulphuraria* [35], may help maintain redox homeostasis by facilitating the conversion of Ni^2+^ to metallic Ni.

Another notable phenomenon was the upregulation of polyamine (PA) biosynthetic genes (Figure 5, Table S1), which suggests the protective role of these metabolites in *C. merolae* during Ni-oxidative stress. PAs stabilize membranes and metal-binding proteins, potentially reducing intracellular metal accumulation and enhancing stress tolerance [36]. Therefore, the transcriptional upregulation of PAs biosynthesis may result in the stabilization of an enzymatic antioxidant and metal chelation machinery during the mitigation of metal toxicity [37]. Our proposed PA biosynthesis pathway suggests arginine, rather than ornithine, as the precursor for putrescin [38] (Figure 5). The upregulation of enzymes such as ornithine carbamoyl transferase and ornithine/arginine decarboxylase (OAD) compensates for the downregulation of ornithine decarboxylase (ODC), while methionine adenosyltransferase (MAT) and S-adenosylmethionine decarboxylase (SAMDC), together with PAPT (putrescine aminopropyl transferase) and SPMS (spermine synthase), promote spermidine (Spd) and spermine (Spm) synthesis. Both PAs are involved in the amelioration of the oxidative stress response [39], inducing the expression of several heat-shock proteins (HSPs) [40], increasing the biosynthesis of the photosynthetic pigments (chlorophyll and carotenoids) [41], and protecting the structural and functional integrity of higher plant thylakoid membranes and photosynthetic apparatus under heavy metal stress [42]. This upregulation is consistent with the increased photosynthetic pigment content and thylakoid membrane stability in Ni-adapted *C. merolae* cells [22].

Interestingly, genes related to peroxisomal function were downregulated during Ni adaptation. Peroxisomes, which manage ROS and lipid metabolism, showed a downregulation of genes related to structural and functional roles (see data for days 5 and 10, Table S1), suggesting a metabolic shift towards energy-saving pathways. This indicates a redirection of resources from energy-intensive processes like membrane biogenesis and lipid metabolism to essential functions such as DNA replication and ROS detoxification.

In summary, the upregulation of PAs biosynthesis genes and key ROS detoxification components suggests that these pathways contribute to the previously observed dynamic amelioration of ROS levels in Ni-exposed *C. merolae* cells [22]. On the other hand, the downregulation of peroxisomal genes points to the induction of an ‘energy-saving mode’ during the Ni adaptation of *C. merolae*, whereby diverting the metabolic resources occurs towards crucial cell survival pathways such as DNA replication, transcription, protein quality control, and heavy metal-induced ROS detoxification.

### 2.4. Protein Synthesis, Folding, and Degradation

Heavy metals exert adverse effects on multiple cellular components, including proteins, through direct interactions or ROS-induced oxidative stress. Cells respond by refolding, removing, and resynthesizing damaged proteins, processes regulated via heat shock proteins (HSPs) [43]. HSPs protect against heavy metal stress by preventing protein aggregation and priming damaged/misfolded proteins for degradation [44].

In this work, we observed the upregulation of several HSPs in 3 mM Ni-adapted *C. merolae* cells (Figure S1, Table S1), including HSP70 (*CMP145C*), which aids in protein refolding and prevent aggregation, thus enhancing heavy metal tolerance [44,45]. HSP70 functions in concert with the co-chaperone DnaJ, which was also upregulated in Ni-treated cells (Table S1). Other upregulated HSPs at 3 mM Ni include nucleotide-exchange factors GrpE (*CMT423C*, *CML088C*), HSP110 (*CMS343C*), HSP90 (*CMA061C*, *CMQ224C*), which stabilizes intracellular proteins [43], and CCT protein (chaperonin containing TCP-1 complex), which is involved in actin and tubulin production [46]. The most broadly upregulated HSP transcript corresponds to the small HSP20 (sHSP20, *CMJ101C*) which was upregulated for all 3 mM Ni timepoints and for days 5 and 10 for 1 mM Ni-treated cells. This HSP prevents protein aggregation [43], as shown for *Tigriopus japonicus* under different metal stress [47].

As heavy metal stress often results in protein damage, it trigger both protein degradation and enhanced translation to biosynthesize properly folded proteins. Indeed, in 3 mM Ni-adapted cells, we observed a massive transcriptional upregulation of ribosomal proteins, including 26 and 38 transcripts for the small ribosomal (40S) and the large (60S) ribosomal subunits, respectively (Figure S1, Table S1). However, the transcripts for ribosomal subunits encoded in the chloroplast genome were downregulated in both 1 and 3 mM Ni-exposed cells (Table S1).

We also observed a differential expression of transcripts coding for proteasome proteins and proteins involved in ubiquitin-meditated proteolysis in Ni-adapted *C. merolae* cells (Figure S1, Table S1). Of the 22 DEGs of proteasomal subunits, most were upregulated in 3 mM Ni-treated cells, particularly on day 10. On the other hand, the transcriptional regulation of ubiquitin–proteasome pathway components is more complex, with upregulation of the E1-ubiquitin activation enzyme and varying regulation of E2 and E3 components involved in protein degradation.

In summary, our transcriptomic data suggest a high demand for chaperonin components required for proper protein folding and the activation of energy-saving metabolic pathways in *C. merolae* cells exposed to Ni stress, as demonstrated by the upregulation of the cytosolic ribosomal subunits and downregulation of the chloroplast-localized counterparts. The upregulation of proteasomal subunit genes and the mixed regulation of ubiquitin-mediated proteolysis transcripts indicate that, in *C. merolae*, protein degradation occurs mainly through ubiquitin-independent proteolysis. Moreover, most transcripts related to protein quality control are differentially expressed in 3 mM Ni cells on day 10, likely coinciding with the metabolic switch to the energy-saving mode.

### 2.5. Lipid Metabolism

Oxidative stress, triggered by heavy metals, leads to lipid peroxidation, which affects membrane properties (fluidity and permeability) and disrupts cell organization and function, as observed for Ni-exposed *C. merolae* cells [22].

In this study, we observed the upregulation of enzymes involved in fatty acid (FA) elongation in the endoplasmic reticulum (ER) (e.g., KCS, 3-ketoacyl-CoA synthase, *CMD118C*; KCR, 3-ketoacyl-CoA reductase, and *CMK172C*; and HCD, 3-hydroxyacyl-CoA dehydratase, and *CMR006C*). However, ACCase (acetyl-CoA carboxylase), which catalyzes the first step of acetyl-CoA conversion into malonyl-CoA, was downregulated both in the cytosol (*CMM188C*) and in the chloroplast (AccA, *CMV056C*; AccD, *CMV207C*). The activity of these enzymes is currently unknown and will be the subject of a future investigation.

The desaturation of fatty acids in phosphatidylcholine, primarily occurring in the ER of *C. merolae* [48], was upregulated in 3 mM Ni-treated cells on day 10 (see *CMK291C*, *CMP111C* and *CMR130C* in Table S1). Similarly, transcripts for enzymes involved in phosphatidylinositol biosynthesis were upregulated (*CME109C*, *CMJ134C* and *CMM125C*, see Table S1). In contrast, transcripts encoding enzymes involved in triacylglycerol (TAG) biosynthesis in the ER (*CMR054C*, *CMN061C* and *CMQ199C*) and those involved in glycolipids and phosphatidylglycerol biosynthesis in the plastid (*CMF185C*, *CMM311C*, *CMR015C* and *CMV121C*) were downregulated (Table S1). The *CMV121C* enzyme catalyzes the conversion of monogalactosyldiacylglycerol (MGDG) into digalactosyldiacylglycerol (DGDG), the two main lipid components of the thylakoid membranes. Beta-oxidation of fatty acids (*CML080C*, *CMC137C* and *CMA042C*) was also downregulated at the transcriptional level in Ni-adapted cells (Table S1).

In conclusion, the remodeling of lipid metabolic transcriptome in *C. merolae* cells exposed to Ni constitutes an adaptative response: the upregulation of FA elongation and desaturation may optimize membrane properties such as permeability and fluidity. On the other hand, the downregulation of TAG, glycolipid, and phosphatidylglycerol biosynthesis likely forms an energy-preserving strategy under Ni stress, redirecting resources to essential survival pathways. In a broader context, changes in lipid metabolism may also contribute to stress responses, whereby altered lipid composition triggers the synthesis of antioxidant molecules to counteract ROS-induced oxidative stress [49].

### 2.6. Energy Metabolism

**Figure 6:**
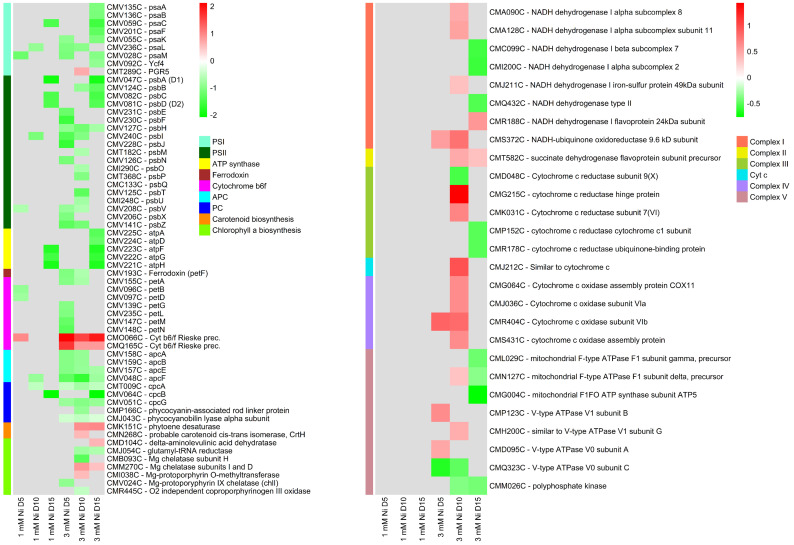
Transcriptomic regulation in *C. merolae* related to molecular components involved in cellular energy metabolism. On the left, a heatmap of DEGs of the photosynthetic apparatus or involved in pigment biosynthesis. On the right, a heatmap of DEGs involved in oxidative phosphorylation in the mitochondria. Data were obtained from three biological replicas (*n* = 3).

Microalgae exposed to environmental stress, such as heavy metals, can redirect energy resources towards repair, maintenance, and defense. Heavy metals affect the photosynthetic apparatus at many levels: directly, by destabilizing photosystem II (PSII) complex and associated chlorophyll molecules, and indirectly via oxidative stress [4]. In *C. merolae* cells exposed to Ni, a general downregulation of the photosynthetic components was observed (Figure 6), including transcripts of photosystem I (PSI), PSII, ATP synthase, ferredoxin, light-harvesting subunits Lhcr, and cytochromes. This downregulation is an adaptive response in photosynthetic organisms under stress, optimizing energy usage while protecting photosynthetic components and avoiding energy imbalance [50,51]. In fact, phototrophs invest energy in immediate adaptive responses necessary for cell survival without losing photosynthetic capacity, thanks to the slow turnover of the photosynthetic proteins [50]. Despite the downregulation of most of the photosynthetic genes, *C. merolae* exposed to 3 mM Ni maintained similar photosynthetic efficiency of both PSI and PSII as the untreated control [22], possibly because the production and reassembly of functional photosynthetic proteins are not closely regulated via transcription [52].

Among several downregulated photosynthetic transcripts, the PGR5 transcript was upregulated at 3 mM Ni. This protein is involved in cyclic electron flow around PSI and in maintaining redox balance of the photosynthetic electron transport. Additionally, the upregulation of two precursors of the iron–sulfur subunit of cytochrome *b*_6_*f* suggests a possible adjustment in the photosynthetic machinery to optimize light energy capture and utilization under Ni stress.

Previously, we showed that Ni exposure affected the total pigment content in *C. merolae*, with the lowest levels measured between days 5 and 10 [22]. However, a complete recovery was observed in 3 mM Ni-treated cells, which can be associated with the upregulation of chlorophyll *a* and carotenoid biosynthetic pathways observed in the present study on days 10 and 15 and with the upregulation of previously discussed PAs.

**Figure 7:**
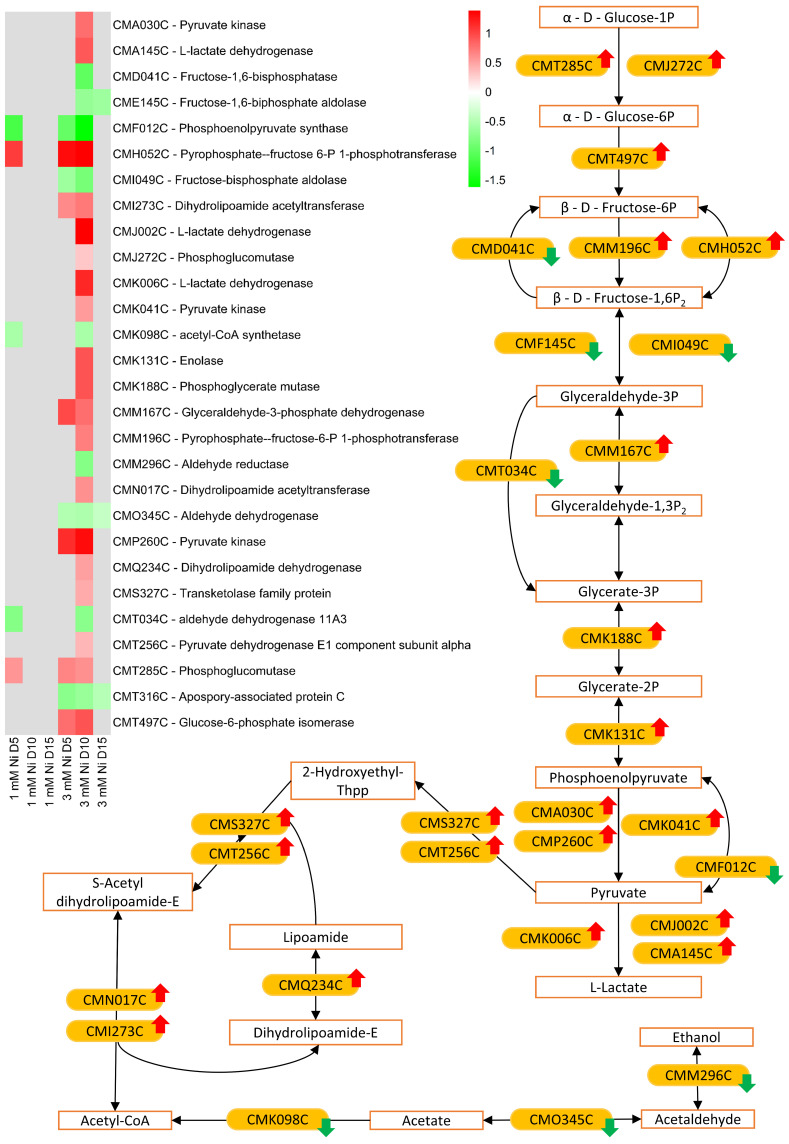
Transcriptomic regulation of glycolysis pathway in *C. merolae* cells exposed to Ni. The up- and downregulation of transcripts involved in glycolysis pathway is depicted with red and green arrows, respectively. The same color coding is used for DEGs in the heatmap (left) for days 1, 5, and 15. Data were obtained from three biological replicas (*n* = 3).

Our data show that, upon Ni exposure, the glycolysis pathway is upregulated, generally on day 10 of 3 mM Ni treatment (Figure 7), alongside the tricarboxylic acid (TCA) cycle and part of oxidative phosphorylation (Figure 6). Glycolysis rapidly generates metabolic energy through substrate-level phosphorylation, producing ATP and NADH, both essential for oxidative phosphorylation. Glycolysis also provides precursors to the biosynthesis of stress-related metabolites and osmoprotectants [53]. Pyruvate, the end product of glycolysis, can enter the TCA cycle, where it is oxidized to produce NADH and FADH2, used in the oxidative phosphorylation for ATP production.

Upon stress, microalgae may increase mitochondrial respiration as an alternative means of ATP production, in particular, if the photosynthetic apparatus is compromised. Given *C. merolae*’s high photosynthetic efficiency under Ni exposure, the upregulation of alternative energy pathways—especially on day 10, when metabolic recovery is postulated to begin (Figure 1 and [22])—suggests that these processes may supply additional energy required to meet increased metabolic demands beyond what photosynthesis alone can provide.

### 2.7. Metal Transport

In plants and algae, metal transporters are fundamental to maintaining metal homeostasis. In *C. merolae*, the characterization of metal transporters, including their sub-cellular localization and possible function, has been predicted only by bioinformatic analysis [54]. Among the different metal transporters, by far, the ATP-binding cassette (ABC) transporters represent one of the largest and most ancient protein superfamilies found in all living organisms. These are molecular machines that are capable of coupling ATP binding, hydrolysis, and phosphate release to the translocation of diverse substrates across membranes. The vast array of substrates of the ABC transporters range from vitamins, lipids, and ions to peptides, proteins, polysaccharides, and xenobiotics [55]. Based on structural similarities, ABC transporters can be classified into several subfamilies, and of these, only two subfamilies have been demonstrated to be directly involved in metal transport: multidrug resistance-associated proteins (MRPs) and heavy metal tolerance/ABC transporters located in the mitochondrial and vacuolar membranes (ATM/HMT). The members of the latter subfamily are involved in transport of heavy metal–phytochelatin complexes from the cytoplasm into the vacuole [56].

In this study, transcripts of the ABC transporters are downregulated in 3 mM Ni cells on day 10 (Table S1), including CmMRP1 (*CMN251C*) and CmATM/HMT-1 (*CMN105C*) transcripts proposed to transport metal cations from the cytosol to the vacuole and iron from mitochondrion to cytoplasm, respectively. Other members of the ABC transporter family seem to retain the same transcript levels as the untreated control at various timepoints and Ni concentrations applied to the *C. merolae* cells.

Apart from the ABC transporters, metal homeostasis is regulated by members of the large and ubiquitous cation diffusion facilitator (CDF) family. Members of this family function as transmembrane divalent d-block metal cation exporters facilitating the efflux of transition metal cations from cytoplasm to subcellular compartments or outside the cells. In this study, we identified the downregulation of the CmMTP2 (*CMC075C*) member of the CDF family on day 5 in both 1 and 3 mM Ni-treated cells, and on day 10 only in 3 mM Ni cells.

In contrast, the CmZIP1 (*CMS155C*) and CmZIP2 (*CMG102C*) members of the ZIP family, also involved in metal homeostasis, are upregulated at all the timepoints for 3 mM Ni-treated cells, and in the case of CmZIP1 also upregulated on day 5 for 1 mM Ni-treated cells.

The FTR, NRMAP, and IREG1 families mainly regulate iron homeostasis using a proton gradient to transport iron and other divalent cations throughout the cells [54]. Our analysis shows that this group of transporters is differentially regulated in *C. merolae*. CmFTR2 (*CML004C*) and CmNRAMP2 (*CML262C*) are significantly upregulated at all timepoints in 3 mM Ni-treated cells and also on day 5 in 1 mM Ni-adapted cells. In contrast, CmFTR4 (*CMN003C*), CmNRAMP1 (*CMJ138C*), and CmIREG1 (*CMG212C*) are downregulated on day 10 and CmNRAMP1 also on day 5 in 3 mM Ni-treated cells.

Amongst the DEGs, we identified Cu transporter CmCOPT1 (*CMS307C*, upregulated on day 10 in 3 mM Ni), Ca/Mn transporter Ccc1p (*CMT466C*, downregulated on day 5 and 10 in 3 mM Ni), and Na transporter (*CML032C*, upregulated on day 5 in 1 mM Ni and in all timepoints of 3 mM Ni). Interestingly, a functional protein domain analysis of the Na-dependent transporter showed that it belongs to the ACR3 family of arsenite permeases which bestow resistance to arsenic by its extrusion from the cells [57], highlighting the versatility of this transporters group. Lastly, the transcript for periplasmic protein p19 (*CMG092C*) is upregulated on days 5, 10, and 15 in 3 mM Ni-exposed cells and on day 5 for 1 mM Ni-treated cells. This protein belongs to the Tp34-type periplasmic metal-binding protein family, functioning with the importer FTR1 and playing a key role in metal sequestration [58].

The transcriptomic data from this study do not clarify the previously reported lack of Ni accumulation inside *C. merolae* cells [22] or whether the transcriptional regulation confers the high metal tolerance, as differentially regulated DEGs were observed within the same family and with the same predicted cellular localization. Nevertheless, the upregulation of CmFTR2, CmZIP1, and CmZIP2, CmNRAMP2, the Na-dependent transporter and the periplasmic protein p19 throughout various timepoints highlights their possible role in maintaining metal homeostasis under heavy metal stress in *C. merolae* cells, perhaps via other metal cation transport systems.

### 2.8. Other Molecular Components

**Figure 8:**
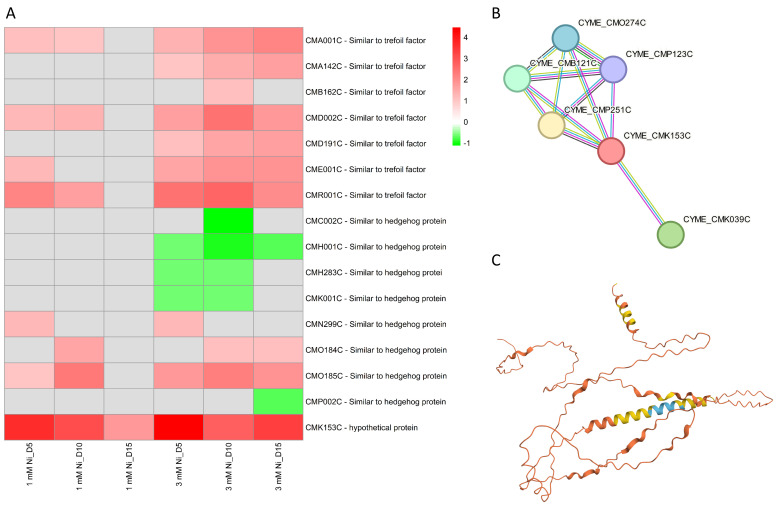
Transcriptomic analysis of ubiquitous DEGs in Ni-treated *C. merolae* cells. (**A**) Heatmap of DEGs involved in various signaling pathways. (**B**) Protein–protein interaction scheme obtained from the STRING analysis of the hypothetical protein *CMK153C*. (**C**) Structure of the *CMK153C* protein as predicted via the AlphaFold webtool (Jumper et al., 2021 [59]). The different color of the protein structure represents the per-residue model confidence score (pLDDT): dark blue—very high (pLDDT > 90), light blue—high (90 > pLDDT > 70), yellow—low (70 > pLDDT > 50) and orange—very low (pLDDT < 50). Data were obtained from three biological replicas (*n* = 3).

Several DEGs coding for trefoil factors proteins were found in this study to be upregulated in both 1 and 3 mM Ni-treated *C. merolae* cells, with upregulation observed across all timepoints (Figure 8A). The function of these proteins is still unknown, but a functional analysis of their protein domains and BLAST (version 2.15.0) analysis reveals the presence of a P-type trefoil domain and a long (~500 aa) Kelch-type β-propeller domain, suggesting a putative protein binding function or similarity to signaling Hedgehog proteins. Many of these proteins were significantly up- or downregulated, implying their role in the fine-tuning of cellular homeostasis during the Ni adaptation of *C. merolae*.

An intriguing observation concerned the transcriptional regulation of the hypothetical protein *CMK153C*, the only transcript that was upregulated at all timepoints in both 1 and 3 mM Ni-exposed cells. A bioinformatic analysis provides clues as to the possible function of this protein: structure prediction, sequence functional analysis, and amino acid alignment suggest a transmembrane nature, with a large extracellular region and two transmembrane α-helical regions (Figure 8C). The hydrophobic N-terminal region of *CMK153C* shares similarities with several transmembrane channel/gate proteins. Moreover, protein–protein interaction analysis (Figure 8B) shows its connection with V-ATPases (*CMO274C*, *CMP123C*), known for pH regulation and proton transport across cellular membrane through ATP hydrolysis; Gtr1/RagA G protein (*CMB121C*, *CMP251C*), important for cell growth, nutrient sensing, and signaling, and the photoregulatory zinc-finger protein COP1 (*CMK039C*), involved in photoprotection and abiotic stress signaling [60]. Given the possible involvement of *CMK153C* in light signaling, nutrient sensing, and ion homeostasis, this transmembrane protein may participate in the cellular responses to metal stress possibly with a role in metal transport. Furthermore, the combination of transmembrane localization and interactions with membrane-bound proteins suggests its role in mediating cell membrane signaling pathways. These hypotheses will be verified in future work.

In general, similar DEG categories were observed after the Ni exposure of other organisms such as higher plants [61,62] or bacteria [63]. Nickel had a significant effect on cell-wall and microtubule organization [61], metal transport/export and homeostasis [61,62,63], DNA repair and epigenetic modifications [62], and photosynthesis [64]. This suggests that, while Ni exposure affects similar categories of genes across taxa, the specific genes and the extent of their differential expression vary, reflecting species-specific adaptative responses to this heavy metal.

## 3. Materials and Methods

### 3.1. Culture Conditions and Experimental Design

*C. merolae* strain NIES-3377 was obtained from the Microbial Culture Collection at the National Institute for Environmental Studies (Tsukuba, Japan (NIES Collection)). The cell culture was cultivated in a Panasonic^®^ Plant Growth Chamber (FL40SS-ENW/37H, Sakata, Japan) using a modified 2× Allen medium [65]. Cell suspensions (125 mL) were grown at pH 2.5, in continuous white light of 90 µE m^−2^ s^−1^ intensity (400–700 nm), with gentle shaking (110 rpm), at 42 °C. These conditions were maintained throughout the experiment. The nickel source used for the stock solution preparation was nickel sulfate hexahydrate (NiSO_4_ × 6(H_2_O)) dissolved in MilliQ water.

At the onset of each experiment, the stock *C. merolae* inoculum (NiSO_4_ × 6 (H_2_O)) and an adequate volume of the modified 2× Allen culture medium were mixed to reach the cell suspension optical density (OD) at 750 nm of 0.15. Samples (10 mL) (3 independent biological replicas, *n* = 3) were collected at given timepoints up to 15 days and then were subjected to further analyses.

### 3.2. Cell Growth Measurements

Cell growth was measured as cell density, expressed as the cell number (number of cells × mL^−1^), and analyzed by counting the number of cells using a Burker Hemocytometer Counting Chamber (Glaswarenfabrik Karl Hecht, Sondheim vor der Rhön, Germany) and an Olympus CX41 light microscope (Olympus, Tokyo, Japan). Based on the cell density, only *C. merolae* control cultures and cultures exposed to 1 and 3 mM NiSO_4_ were selected and considered for transcriptomic analysis.

### 3.3. Total RNA Extraction

Total RNA was isolated from untreated *C. merolae* cells and cells exposed to 1 and 3 mM Ni. A volume of 10 mL of microalgae cell suspension was collected on days 0, 5, 10, and 15 of each experiment. Next, microalgae cell suspensions were centrifuged at 2200 g for 15 min at 4 °C. Then, the samples were frozen and stored at −80 °C. RNA extraction was performed using TRIzol reagent (Invitrogen, Carlsbad, CA, USA). RNA concentration was measured using a DeNovix DS-11 spectrophotometer (Wilmington, DE, USA) and the purity was assessed through the evaluation of A_260_/A_280_ and A_260_/A_230_ absorbance ratios. In parallel, RNA integrity and quality were assessed with a Bioanalyzer (Agilent Technologies, Inc., Santa Clara, CA, USA). Extracted RNA was stored at −80 °C until further analysis.

### 3.4. Construction of RNA-Seq Libraries and Sequencing

After the RNA quality analysis was performed using Agilent RNA 6000 Pico Kit (Agilent), it was determined that five samples needed additional RNA purification using KAPA Pure Beads (KAPA Biosystems, Wilmington, MA, USA). Enrichment was performed using NEBNext Poly (A) mRNA Magnetic Isolation Module (New England Biolabs, Ipswich, MA, USA). Libraries (mRNA-seq) were constructed using 1 μg of material according to the KAPA mRNA Hyperprep (KAPA Biosystems, Wilmington, MA, USA) procedure with KAPA Unique Dual Indexes 96 Plate (KAPA Biosystems, Wilmington, MA, USA). Subsequently, fragmentation was conducted within 5 min at 94 °C followed by 10 cycles of PCR enrichment. The quality of obtained libraries was analyzed using Agilent TapeStation 2200 with High Sensitivity D1000 ScreenTape (Agilent) and High Sensitivity D1000 Reagents (Agilent). Finally, the quantitative analysis of libraries was measured via qPCR with the KAPA Library Quantification kit (KAPA Biosciences, Wilmington, MA, USA, cat. no. KK48sun), according to the manufacturer’s protocol. Sequencing was performed on Illumina NovaSeq 6000 with NovaSeq 6000 S1 Reagent Kit (200 cycles) (Illumina, cat. no. 20012864, Neutral Bay, New South Wales), using 100-bp paired-end reads following standard operating procedures and 1% PhiX control library addition. As a result, high-quality data were obtained (more than 93% of data of quality over Phred Score Q37) in a quantity of 4.3–7.3 MR/sample.

### 3.5. Differentially Expressed Genes Analysis

Raw sequences were trimmed according to quality using Trimmomatic [66] (version 0.39) using default parameters, except MINLEN, which was set to 50. Trimmed sequences were mapped to *C. merolae* reference genome provided via ENSEMBL (version ASM9120v1) using Hisat2 [67] with default parameters. Optical duplicates were removed using MarkDuplicates tool from GATK [68] package (version 4.2.3.0) with default parameters except OPTICAL_DUPLICATE_PIXEL_DISTANCE set to 12,000. Reads that failed to map to the reference were extracted using Samtools [69] and mapped to Silva meta-database of rRNA sequences [70] (version 119) with Sortmerna [71] (version 2.1b) using “–best 1” option. Mapped reads were associated with transcripts from ASM9120v1 database [72] (Ensembl, version 54) using HTSeq-count [73] (version 2.0.1) with default parameters except –stranded set to “reverse”. DDEGs were selected using DESeq2 package [74] (version 1.26.1). To consider whether one specific gene was differentially expressed, two main criteria were followed: fold change (FC) > 1 or <1, either for an upregulated or downregulated gene, respectively, and an adjusted *p*-value < 0.05. Fold change was corrected using the lfcShrink function from DESeq2 package (option: type = “normal”).

### 3.6. Gene Ontology and KEGG Pathway Enrichment Analysis

Once the DEGs under nickel exposure of *C. merolae* microalgal cells were determined, a gene ontology (GO) and *Kyoto Encyclopedia of Genes and Genomes* (KEGG) pathway analysis was performed to determine the possible cellular function or metabolic pathway to which each DEG corresponds. The DEGs were then divided into three different categories, such as molecular function (MF), biological process (BP), and cellular component (CC). As in the case of DEGs, fold change (FC) > 1 or <1, either for an up- or downregulated gene, respectively, and an adjusted *p*-value < 0.05 were considered significant and used in this analysis. A K-means clustering analysis, among others, was generated and conducted via iDEP 9.6 (integrated differential expression and pathway analysis) webtool (http://bioinformatics.sdstate.edu/idep96/ (accessed on 30 November 2023)) [75].

### 3.7. Statistical and Bioinformatic Analysis

For the transcriptomic analysis, three biologically independent replicates of untreated and Ni-treated cells were analyzed. Additionally, group diagrams were used to display which specific differentially expressed gen was unique or shared between the different nickel treatments and timepoints. On the other hand, volcano plots and heatmaps were also generated and created using R, and principal component analysis (PCA) was carried out to determine the reproducibility between the three biologically independent replicates. In the case of up- or downregulated transcripts whose function was unknown or unclear, further bioinformatics analyses were conducted to elucidate their potential roles and functional significance. The bioinformatic resources of InterPro [76] for a comprehensive and integrated analysis of protein sequences and domains, BLAST [77] for the comparison of primary biological sequence, and STRING [78] for the analysis of protein–protein interactions, and AlphaFold [59] were employed.

## 4. Conclusions

This study has described the first global transcriptome analysis of the acido-thermophilic red microalga *C. merolae* in response to extremely high Ni concentrations. It has revealed the possible molecular components and pathways underlying the long-term adaptation of this model extremophile to heavy metal toxicity. The DEG analysis revealed that cells undergo an energy-saving metabolic switch in response to Ni. Metabolic pathways critical for cell survival, such as DNA replication, cell cycle, protein-quality control, and ROS amelioration are transcriptionally upregulated, while energetically costly processes, including assembly of the photosynthetic apparatus and lipid biosynthesis, are downregulated.

Among the most highly upregulated transcripts across all Ni treatments and timepoints, *CMK153C*, likely involved in metal transport, stood out for its consistent and strong induction—reaching a maximum 22-fold increase on day 5 in 3 mM Ni-exposed cells and remaining elevated across all other conditions. *CML262C* and *CMJ101C*, encoding a putative metal transporter and a small heat-shock protein of the HSP20 family, respectively, were also among the most upregulated transcripts, suggesting their key roles in the early Ni stress response. Other notably induced genes, such as *CMR001C* (similar to a trefoil factor), point towards the importance of metal transport, protein folding, and oxidative stress protection as part of the Ni adaptation strategy of *C. merolae*. In contrast, *CMR269C*, coding for a NagB/RpiA/CoA transferase-like superfamily protein, was the most consistently downregulated transcript across all the Ni exposure conditions. *CMM104C* and *CMO314C*, encoding an insulinase (peptidase family M16) and a PSII stability/assembly factor, respectively, were among the most repressed genes, each showing over 0.85-fold downregulation compared to the untreated control.

Our study shows that the upregulation of molecular components involved in DNA repair and replication, as well as chromosome condensation and segregation are likely to constitute the high-level control of the downstream metabolic pathways differentially regulated in *C. merolae* cells exposed to extremely high Ni levels. This, in turn, provides the basis for the observed cell growth recovery, whereby the optimized metabolic energy expenditure is used for cell survival. Our comprehensive transcriptomic analysis lays the foundation for future directions in multi-omic fundamental studies of stress adaptation in extremophilic microorganisms. Last but not least, the differentially regulated genes identified in this study provide important clues on the possible molecular targets for the development of rational strategies for heavy metal bioremediation in aquatic environments.

## Data Availability

The data discussed in this publication have been deposited into NCBI’s Gene Expression Omnibus [79] and are accessible through GEO Series accession number GSE284716 (https://www.ncbi.nlm.nih.gov/geo/query/acc.cgi?acc=GSE284716 (accessed on 19 December 2024)).

## Supplementary Materials

The following supporting information can be downloaded at https://www.mdpi.com/article/10.3390/ijms26104813/s1.
