# Supplementary material for: Transcriptomics Reveals an Energy-Saving Metabolic Switch in an Extremophilic Red Microalga Cyanidioschyzon merolae Under Nickel Stress
Source: Int J Mol Sci. 2025 May 17;26(10):4813. doi: 10.3390/ijms26104813 (PMC12112623; doi:10.3390/ijms26104813)
Supplement: Supplementary file 1 [file ijms-26-04813-s001.zip › ijms-3616241-supplementary.pdf]

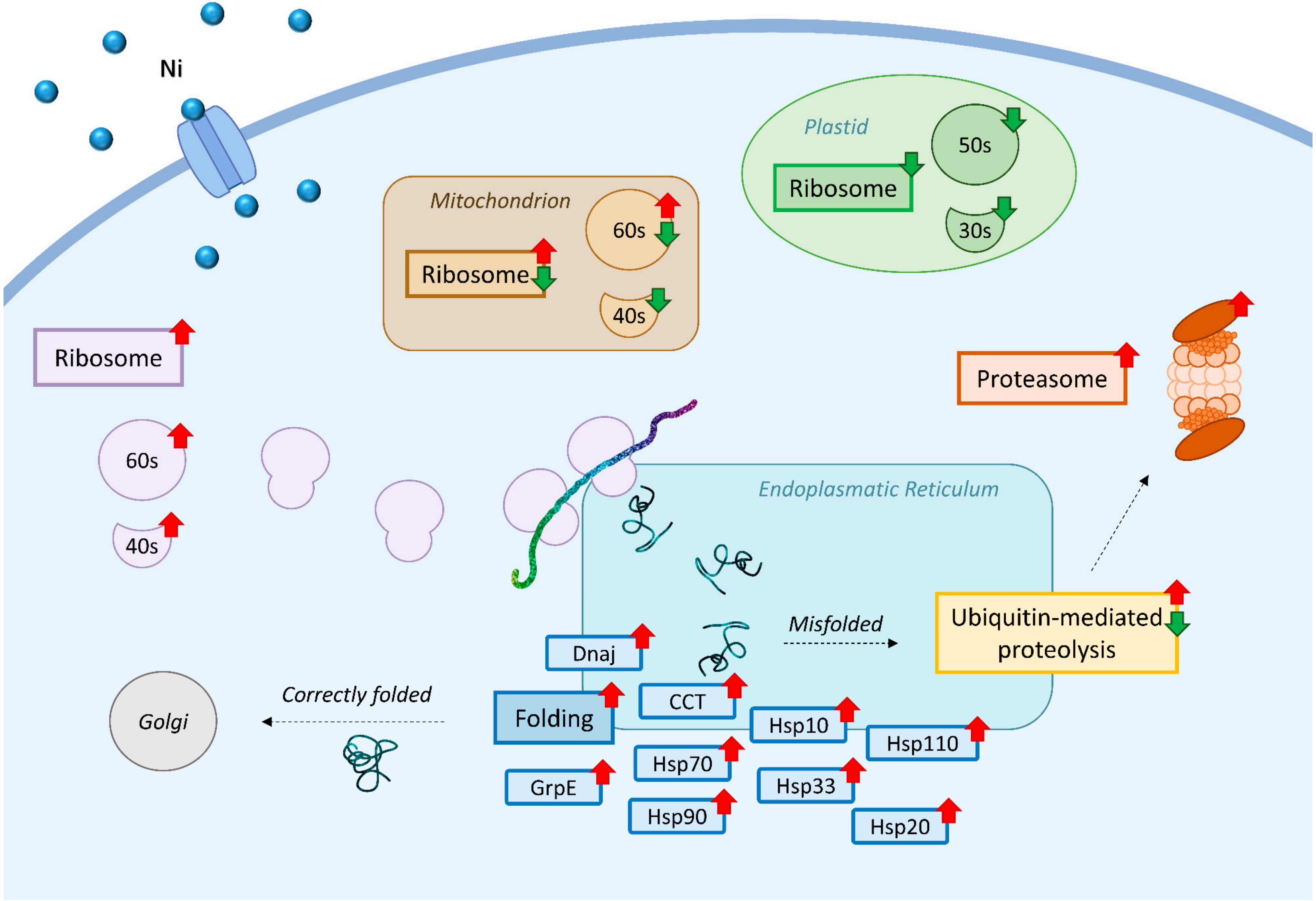
Ni
Mitochondrion
Ribosome
60s
40s
Plastid
Ribosome
50s
30s
Ribosome
60s
40s
Proteasome
Endoplasmatic Reticulum
Misfolded
Ubiquitin-mediated proteolysis
Dnaj
Folding
CCT
Hsp10
Hsp110
Hsp70
GrpE
Hsp33
Hsp90
Hsp20
Golgi
Correctly folded

Figure S1. Transcriptomic regulation of protein synthesis, folding and degradation. The diagrammatic representation of the up- and downregulation of molecular components and pathways for protein synthesis, folding, and degradation in *C. merolae* cells exposed to high Ni levels. Transcriptional remodelling is shown for the cytoplasm, mitochondrion, plastid, and endoplasmic reticulum.

Table S1. Differentially regulated genes of various functionalities. The table presents all the DEGs discussed in the main manuscript, categorized by their biological functions: protein quality control machinery, lipid-fatty acid metabolism, polyamine metabolism, metal transporter and complexing, ROS detoxification machinery, and energy metabolism. For each DEG, the gene name, accession number, and colour-coded expression pattern are provided for samples of *C. merolae* cells treated with 1 and 3 mM Ni. Upregulated and downregulated genes are highlighted in shades of red and green, respectively. The data are based on three biological replicas ($n = 3$).

Module	Gene	Accession n°	1 mM Ni			3 mM Ni		
			Day 5	Day 10	Day 15	Day 5	Day 10	Day 15
DNA repair, recombination, regulation and replication	alpha-tubulin	CMT504C						
	alpha-tubulin	CMT504C						
	ATP-dependent DNA helicase RecQ	CMA093C						
	bacterial DNA polymerase I	CMT462C						
	beta-tubulin	CMN263C						
	cell division cycle protein cdc23	CML190C						
	centromere protein E, CENP-E protein	CMQ429C						
	chromosome assembly complex Condensin I, subunit G	CMS422C						
	chromosome assembly complex Condensin II, subunit D3	CMQ236C						
	chromosome assembly complex Condensin II, subunit G2	CMA089C						
	chromosome assembly complex Condensin II, subunit H2	CMI207C						
	chromosome assembly complex Condensin, core subunit C - SMC4	CME029C						
	chromosome assembly complex Condensin, core subunit E - SMC2	CMG189C						
	cyclin-dependent kinase regulatory subunit	CMA127C						
	DNA excision repair protein ERCC2/XPD	CMP065C						
	DNA gyrase subunit B	CMH166C						

DNA ligase I	CMK235C
DNA polymerase alpha, catalytic subunit	CMI176C
DNA polymerase delta, catalytic subunit	CMN199C
DNA polymerase delta, subunit B	CMB052C
DNA polymerase delta, subunit D	CMT350C
DNA polymerase epsilon, subunit B	CMH082C
DNA polymerase iota, RAD30	CMA062C
DNA primase, large subunit	CMK284C
DNA primase, small subunit	CMO313C
DNA repair protein RAD52	CMS368C
DNA repair protein REV1, deoxycytidyl transferase	CMS282C
DNA repair protein SMC6	CMA066C
DNA replication complex GINS protein Psf2	CMQ381C
DNA replication licensing factor MCM2	CMD025C
DNA replication licensing factor MCM4	CMP356C
DNA replication licensing factor MCM5	CMF173C
DNA replication licensing factor MCM6	CMJ261C
DNA replication licensing factor MCM7	CMR234C
DNA topoisomerase I	CMI252C
excinuclease ABC subunit B (UvrABC system, subunit B)	CMG133C
exonuclease I - endonuclease	CME067C
gamma-tubulin	CMN304C
GINS complex subunit Sld5	CMR406C
histone acetyltransferase HAC108/MYST	CML102C
hypothetical protein	CMB086C
hypothetical protein	CMN041C
hypothetical protein	CMO116C
hypothetical protein - ARID DNA-binding domain	CMS239C
hypothetical protein - E2F/DP family/ Transcription factor DP	CML181C
hypothetical protein - Extended PHD (ePHD) domain	CMR172C
hypothetical protein - DNA replication complex GINS protein PSF1	CMR136C
hypothetical protein - DNA2/NAM7-like helicase	CMK133C

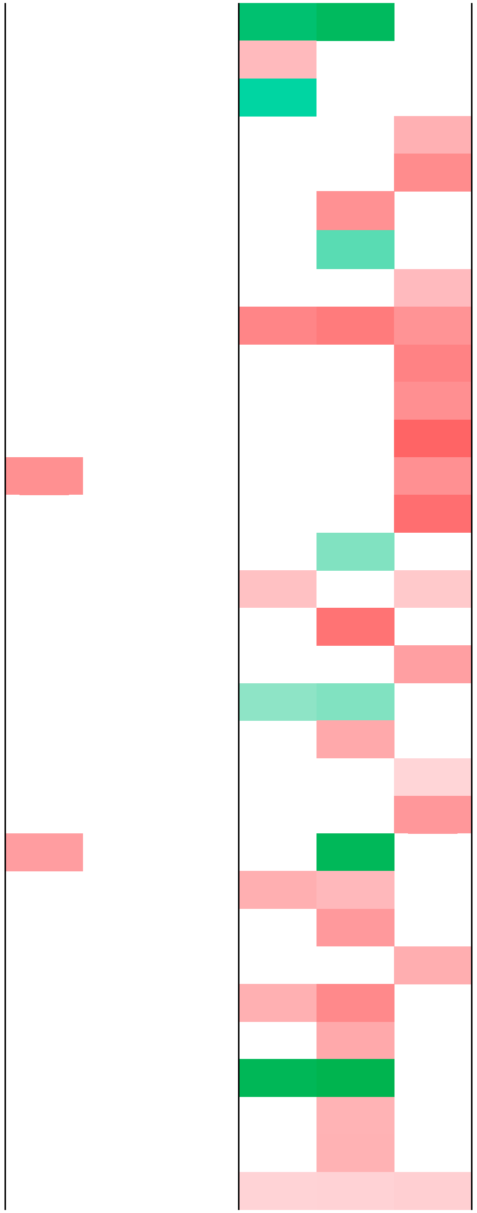

hypothetical protein - E3 SUMO-protein ligase Nse2 (Mms21)	CMP167C
hypothetical protein - Fanconi anaemia protein FANCD	CMN306C
hypothetical protein - GINS complex, subunit Psf3	CMP212C
hypothetical protein - GNAT domain	CMT193C
hypothetical protein - Kinetochore protein Spc25	CMI101C
hypothetical protein - Kinetochore-Ndc80 subunit Spc24	CMG186C
hypothetical protein - Mad1	CMP244C
hypothetical protein - Mitotic spindle checkpoint protein Bub1/Mad3	CMK144C
hypothetical protein - Protein DEK	CMM055C
hypothetical protein - S-adenosyl-L-methionine-dependent methyltransferase ML2640-like	CMN118C
hypothetical protein - spindle and kinetochore-associated protein 1 SKA1	CMI099C
hypothetical protein - Structure-specific endonuclease subunit Slx4	CMI177C
hypothetical protein - TFIIH subunit Tfb1/GTF2H1	CME176C
hypothetical protein - to form a complex with endonuclease activity	CMC115C
hypothetical protein, conserved	CMP244C
hypothetical protein, conserved - SOS response associated peptidase (SRAP)	CMQ063C
hypothetical protein, conserved - Kinetochore protein Ndc80p	CMG078C
hypothetical protein, conserved - RPA-related protein RADX	CMQ139C
kinesin-related protein	CMR497C
kinesin-related protein, C-terminal motor subfamily	CMT097C
mutS family DNA mismatch repair protein MSH4	CMB035C
mutS family DNA mismatch repair protein MSH4	CMK199C
mutS family DNA mismatch repair protein MSH6	CMI229C
probable AAA protein spastin	CMN138C
probable ATP-dependent DNA helicase RecQ	CMH155C
probable chromosome segregation protein CSE1	CMN027C
probable class II DNA photolyase	CMH170C
probable cryptochrome DASH	CMJ130C

probable DNA cytosine-5 -methyltransferase 3	CMR026C
probable DNA mismatch repair protein MutL/Mlh/Pms-like	CMI169C
probable DNA polymerase alpha, subunit B	CMF127C
probable DNA repair protein RadA	CMK251C
probable DNA repair protein SPR18 - Structural maintenance of chromosomes protein 5 - SMC5	CMH246C
probable DNA replication licensing factor MCM2 - MCM9 like	CMO137C
probable flap structure-specific endonuclease 1 - XPG/Rad2 endonuclease	CMG106C
probable G2/mitotic-specific cyclin 1	CMI227C
probable proliferating cell nuclear antigen (PCNA)	CMS101C
probable replication protein A	CMC123C
probable spindle checkpoint protein kinase MPS1	CMB064C
replication factor C subunit 1 (RFC1)	CMR028C
replication factor C subunit 2	CMF110C
similar to anti-silencing factor - Histone chaperone ASF1-like	CMG143C
similar to candidate tumor suppressor - ING family	CMQ358C
similar to cell division cycle protein CDC6	CME082C
similar to cell polarity protein alp13 (MRG)	CMS194C
similar to chromatin accessibility complex 1	CMQ472C
similar to DNA apurinic or apyrimidinic site lyase ARP	CMG142C
similar to DNA replication licensing factor MCM8	CMT087C
similar to DNMT1 associated protein 1	CMN257C
similar to endonuclease III	CMD139C
similar to meiotic nuclear division protein Mnd1	CMG028C
similar to methyl transferase	CMN180C
similar to microtubule-associated protein	CMP130C
similar to microtubule-associated protein, RP/EB family	CMJ156C
similar to nucleosome/chromatin assembly factor C	CMQ367C
similar to origin recognition complex subunit 1	CMS112C
similar to origin recognition complex subunit 4	CMT300C
similar to PCAF histone acetylase complex subunit	CMO046C

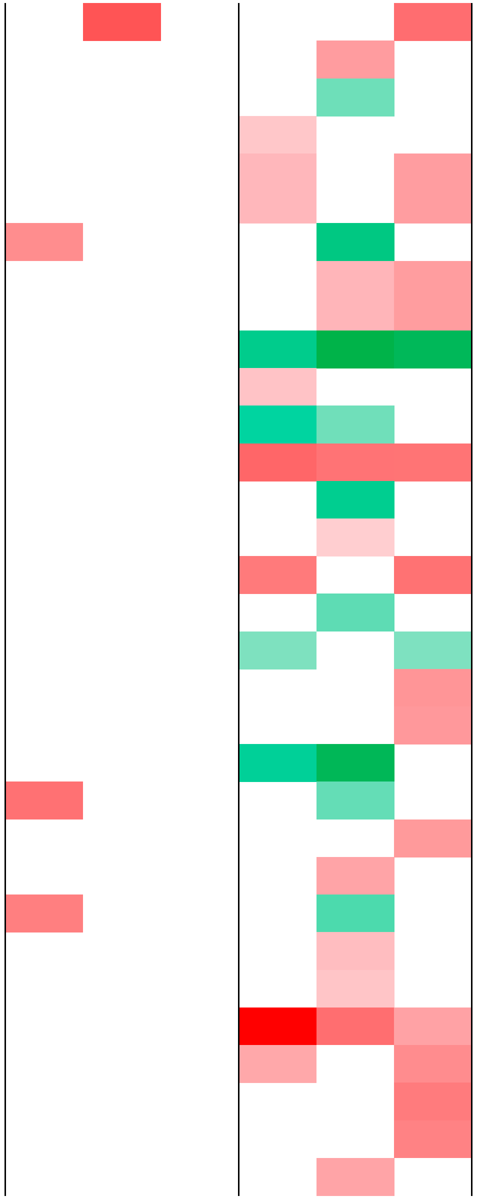

	similar to protein N-methyltransferase - SET domain superfamily	CMT147C
	similar to replication protein A 30kDa (RFA2-like)	CMI291C
	similar to separin	CMD159C
	similar to spindle checkpoint protein Xmad1 - Mad1	CME139C
	similar to spindle pole body associated protein - Kinetochore-Ndc80 subunit Nuf2	CMB012C
	similar to TFIIH subunit SSL1	CMT290C
	similar to UVB-resistance protein UVR8	CMH019C
	Sister chromatid cohesion protein Dcc1	CMN029C
	sister-chromatide cohesion complex Cohesin, subunit SMC1	CMI192C
	sister-chromatide cohesion complex Cohesin, subunit SMC3	CML027C
	spindle checkpoint protein kinase MPS1	CMB108C
	TATA-box binding protein-associated factor 9	CMO165C
	topoisomerase II	CMB013C
	transcription-repair coupling factor	CMC111C
	valosin-containing protein - AAA+ ATPase domain	CMS124C
Polyamine metabolism	ornithine aminotransferase	CML323C
	ornithine carbamoyltransferase	CML214C
	ornithine/arginine decarboxylase	CMI221C
	probable ornithine decarboxylase	CMA052C
	putrescine aminopropyltransferase	CMP329C
	S-adenosylmethionine decarboxylase	CMO012C
	S-adenosylmethionine synthase	CMN227C
	spermine synthase	CMR256C
ROS detoxification machinery	catalase	CMI050C
	cytosolic ascorbate hydrogen peroxidase	CMC150C
	dihydrolipoamide dehydrogenase	CMQ234C
	glutathione reductase	CMC043C
	hypothetical protein, conserved	CMQ019C
	mercuric reductase	CMJ014C
	mitochondrial Mn superoxide dismutase	CMR158C

	Mn superoxide dismutase:	CMT028C
	monodehydroascorbate reductase	CMT414C
	probable peroxisomal biogenesis factor 6	CML252C
	Similar to biotin synthase	CMK049C
	similar to glutathione S-transferase	CMR110C
	Similar to glutathione S-transferase family protein	CMO318C
	similar to microsomal glutathione S-transferase	CMS309C
	similar to peroxisomal membrane protein PEX2	CMD105C
	similar to peroxisomal membrane protein PMP22	CMG125C
	similar to peroxisomal membrane protein PMP22	CMG206C
	similar to peroxisomal membrane protein PMP22	CMS057C
	similar to peroxisomal targeting signal type 1 receptor PEX5	CMK124C
	similar to thioredoxin f	CMN279C
	similar to thioredoxin-like protein	CMT037C
	thioredoxin	CMQ093C
Protein quality control machinery	20S core proteasome subunit alpha 1	CMN208C
	20S core proteasome subunit alpha 2	CML273C
	20S core proteasome subunit alpha 3	CMR302C
	20S core proteasome subunit alpha 6	CME128C
	20S core proteasome subunit alpha 7	CMO197C
	20S core proteasome subunit beta 2	CMK289C
	20S core proteasome subunit beta 4	CMH119C
	20S core proteasome subunit beta 5	CMO149C
	20S core proteasome subunit beta 6	CMT477C
	20S core proteasome subunit beta 7	CMD060C
	26S proteasome ATP-dependent regulatory subunit	CMC114C
	26S proteasome ATP-dependent regulatory subunit	CMI156C
	26S proteasome ATP-dependent regulatory subunit	CMJ186C
	26S proteasome ATP-dependent regulatory subunit	CMK294C
	26S proteasome regulatory subunit RPN1	CMP340C
	26S proteasome regulatory subunit RPN11	CMC013C
	26S proteasome regulatory subunit RPN12	CMJ011C

26S proteasome regulatory subunit RPN2	CMQ283C
26S proteasome regulatory subunit RPN6	CMS045C
26S proteasome regulatory subunit RPN7	CMG170C
26S proteasome regulatory subunit RPN8	CMT431C
26S proteasome regulatory subunit RPN9	CMQ395C
30S ribosomal protein S14 (rps14)	CMV137C
30S ribosomal protein S16 (rps16)	CMV084C
30S ribosomal protein S18 (rps18)	CMV213C
30S ribosomal protein S2 (rps2)	CMV218C
30S ribosomal protein S3 (rps3)	CMV170C
30S ribosomal protein S5 (rps5)	CMV180C
30S ribosomal protein S6 (rps6)	CMV079C
40S ribosomal protein S10	CMJ285C
40S ribosomal protein S11	CMO287C
40S ribosomal protein S14	CMH046C
40S ribosomal protein S15	CMG109C
40S ribosomal protein S15A	CMI202C
40S ribosomal protein S16	CMP007C
40S ribosomal protein S17	CMT402C
40S ribosomal protein S18	CMB004C
40S ribosomal protein S19	CMR148C
40S ribosomal protein S2	CMA082C
40S ribosomal protein S20	CMG057C
40S ribosomal protein S21	CMQ051C
40S ribosomal protein S23	CMI059C
40S ribosomal protein S24	CMA114C
40S ribosomal protein S25	CMM240C
40S ribosomal protein S26	CMN126C
40S ribosomal protein S27	CMT197C
40S ribosomal protein S27A, ubiquitin fusion protein	CMN125C
40S ribosomal protein S3	CMN148C
40S ribosomal protein S3A	CMS189C

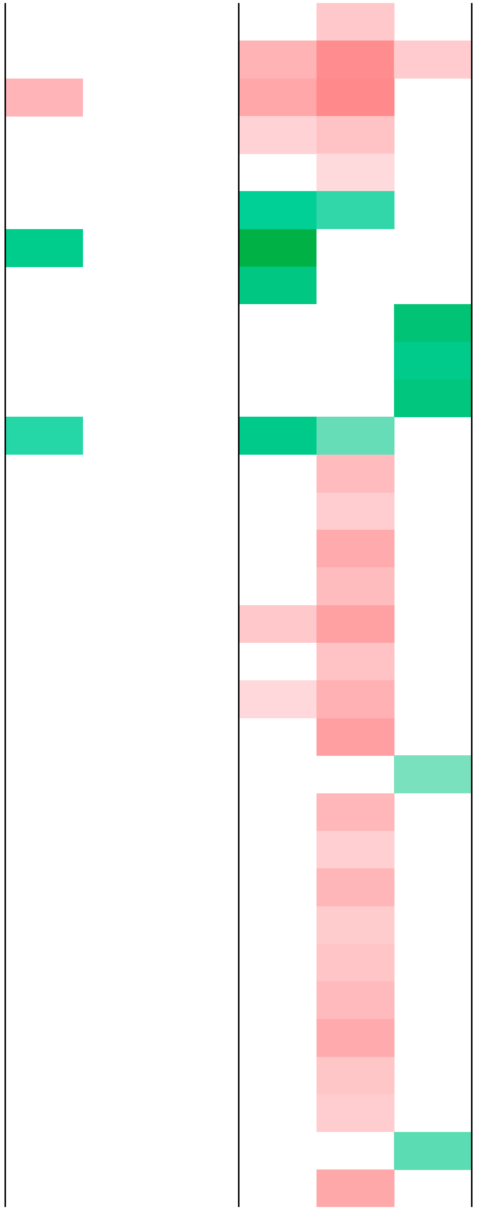

40S ribosomal protein S4	CMH218C
40S ribosomal protein S5	CMT627C
40S ribosomal protein S6	CMT154C
40S ribosomal protein S7	CMA122C
40S ribosomal protein S8	CMT159C
40S ribosomal protein SA	CMT410C
50S ribosomal protein L11 (rpl11)	CMV102C
50S ribosomal protein L14 (rpl14)	CMV174C
50S ribosomal protein L16 (rpl16)	CMV171C
50S ribosomal protein L2 (rpl2)	CMV167C
50S ribosomal protein L20 (rpl20)	CMV151C
50S ribosomal protein L21 (rpl21)	CMV034C
50S ribosomal protein L33 (rpl33)	CMV214C
50S ribosomal protein L34 (rpl34)	CMV072C
60 kDa chaperonin (groEL)	CMV086C
60S acidic ribosomal protein P0	CMQ445C
60S acidic ribosomal protein P1	CMI140C
60S acidic ribosomal protein P2	CMG118C
60S acidic ribosomal protein PO	CMB048C
60S ribosomal protein L10	CMJ109C
60S ribosomal protein L10A	CMP179C
60S ribosomal protein L11	CML196C
60S ribosomal protein L12	CMS284C
60S ribosomal protein L13	CMP006C
60S ribosomal protein L13A	CMR122C
60S ribosomal protein L14	CMH065C
60S ribosomal protein L15	CMJ110C
60S ribosomal protein L17	CMQ463C
60S ribosomal protein L18	CMB018C
60S ribosomal protein L18a	CMO302C
60S ribosomal protein L19	CMJ057C
60S ribosomal protein L22	CMS080C

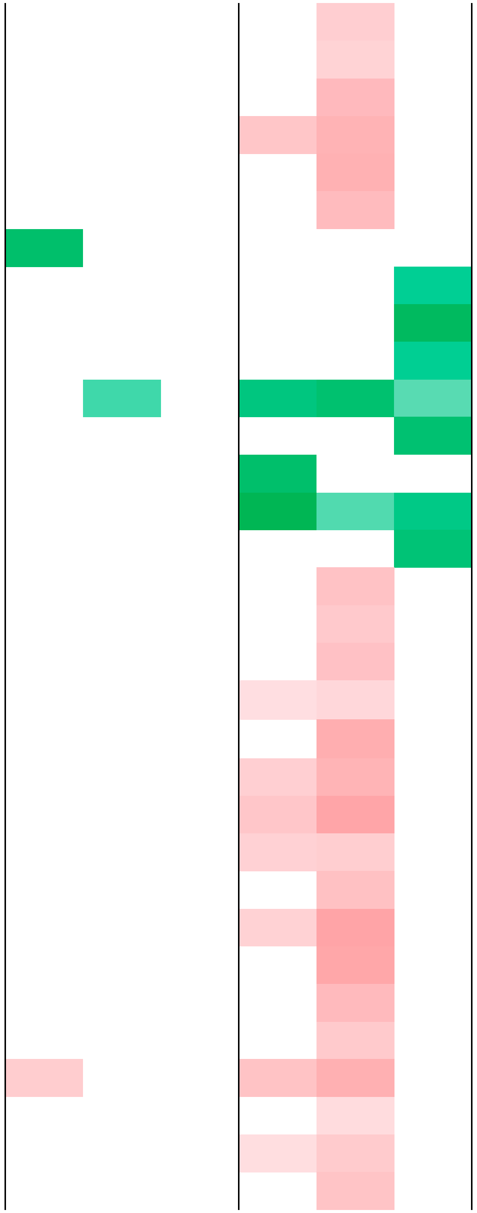

60S ribosomal protein L23A	CMK273C
60S ribosomal protein L24 homolog	CMT160C
60S ribosomal protein L26	CMG157C
60S ribosomal protein L27	CML305C
60S ribosomal protein L27A	CMD140C
60S ribosomal protein L29	CME046C
60S ribosomal protein L3	CMM253C
60S ribosomal protein L32	CMJ289C
60S ribosomal protein L34	CMP012C
60S ribosomal protein L35A	CML304C
60S ribosomal protein L36	CML106C
60S ribosomal protein L37A	CMN315C
60S ribosomal protein L38	CMJ170C
60S ribosomal protein L39	CMQ118C
60S ribosomal protein L4	CMQ293C
60S ribosomal protein L5	CMH071C
60S ribosomal protein L6	CMQ078C
60S ribosomal protein L7	CMO310C
60S ribosomal protein L7A	CML317C
60S ribosomal protein L8	CMR287C
60S ribosomal protein L9	CMC145C
anaphase-promoting complex subunit 2 (Apc2)	CMC102C
ATP-dependent clp protease ATP-binding subunit (clpC)	CMV107C
ATP-dependent Clp protease, ATP-binding subunit ClpB	CMS128C
ATP-dependent Hsl protease ATP-binding subunit HslU, heat shock protein HslU	CMT122C
box C/D snoRNP component Nop58	CMT605C
Cdc20	CMA138C
chaperonin containing TCP1, subunit 1	CMT207C
chaperonin containing TCP1, subunit 2	CMT322C
chaperonin containing TCP1, subunit 3	CMB044C
chaperonin containing TCP1, subunit 5	CML276C

chaperonin containing TCP1, subunit 6a	CME197C
chaperonin containing TCP1, subunit 6a	CMG005C
chaperonin containing TCP1, subunit 7	CMT558C
chaperonin containing TCP1, subunit 8	CMT318C
chloroplast molecular chaperone GrpE	CMT423C
chloroplast ribosomal protein L15 precursor	CMQ292C
chloroplast ribosomal protein S21 precursor	CMB032C
chloroplast translation elongation factor G	CMK150C
chroloplast chaperonin CPN60, precursor	CMB021C
cullin 1	CMT046C
DegP protease	CMM292C
DnaJ homolog, subfamily A	CML030C
DnaK; Hsp70-type chaperone	CMV163C
eukaryotic translation elongation factor 2	CMS428C
eukaryotic translation initiation factor eIF-2B alpha subunit	CMR269C
eukaryotic translation initiation factor eIF-2B delta subunit	CMT047C
eukaryotic translation initiation factor eIF-2B epsilon subunit	CMR397C
eukaryotic translation initiation factor eIF-3 subunit H	CMS354C
eukaryotic translation initiation factor eIF-4F	CMQ348C
eukaryotic translation initiation factor eIF-5A	CMS351C
glutamyl-tRNA amidotransferase subunit A	CMM318C
heat shock protein Hsp70, cytosolic	CMP145C
heat shock protein of Hsp90 family	CMA061C
heat shock protein of Hsp90 family	CMQ224C
hsp90 co-chaperone p23	CMQ190C
hypothetical protein	CMB107C
hypothetical protein	CMM163C
hypothetical protein	CMD103C
hypothetical protein	CMJ088C
hypothetical protein	CMD173C
hypothetical protein	CMO203C
hypothetical protein	CMM121C

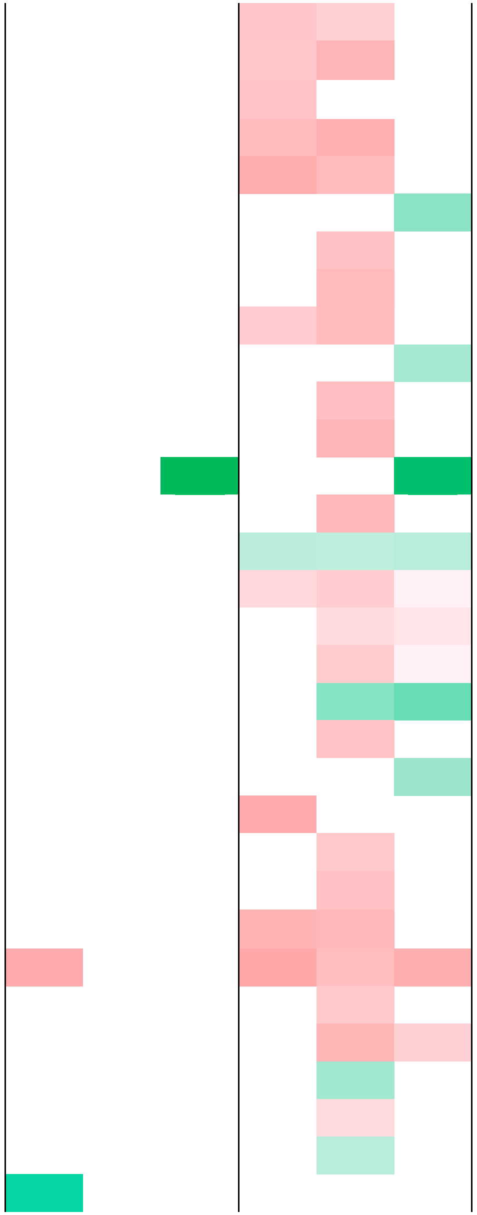

hypothetical protein	CMS402C
hypothetical protein	CMS402C
hypothetical protein	CMA028C
hypothetical protein - HP-VHLBOX	CMD063C
hypothetical protein, conserved	CMP203C
mitochondrial chaperonin hsp10, precursor	CMF129C
mitochondrial ribosomal protein L13 precursor	CMH224C
mitochondrial ribosomal protein L15 precursor	CMC132C
mitochondrial ribosomal protein L17 precursor	CMT508C
mitochondrial ribosomal protein L19 precursor	CME190C
mitochondrial ribosomal protein L21 precursor	CMS047C
mitochondrial ribosomal protein L23 precursor	CMN112C
mitochondrial ribosomal protein L24 precursor	CMI055C
mitochondrial ribosomal protein L30 precursor	CML149C
mitochondrial ribosomal protein L35 precursor	CMN012C
mitochondrial ribosomal protein L36 precursor	CMP301C
mitochondrial ribosomal protein L4 precursor	CMQ421C
mitochondrial ribosomal protein L46	CMH275C
mitochondrial ribosomal protein S15 precursor	CMH066C
mitochondrial ribosomal protein S7, precursor	CMT485C
mitochondrial translation elongation factor G1	CMG196C
molecular chaperone of Hsp110 family	CMS343C
probable ATP-dependent Clp protease regulatory subunit CLPX	CMT045C
probable carboxyl-terminal protease	CMP297C
probable chloroplast chaperonin CPN21, precursor	CMS188C
probable DegP protease precursor	CML090C
probable endoplasmic reticulum oxidoreductin 1-Lbeta	CMC022C
probable eukaryotic translation initiation factor eIF-3 subunit J	CMQ285C
probable eukaryotic translation initiation factor eIF-3 subunit K	CMC106C
probable GTP-binding protein	CMJ037C
probable ribosome biogenesis protein NEP1	CMC063C
probable serine protease	CMD006C

probable trigger factor	CMI148C
probable ubiquitin conjugating enzyme E2	CMR010C
probable ubiquitin fusion degradation protein Ufd4p	CMI198C
probable ubiquitin-conjugating enzyme E2	CMB015C
probable ubiquitin-conjugating enzyme E2	CMG136C
probable zinc protease	CMI130C
protease IV	CMP160C
putative ribosomal protein 3 (ycf65)	CMV085C
serine protease	CML185C
similar to 60S ribosomal subunit biogenesis protein Nip7p	CMN108C
similar to ATP binding protein	CMB065C
similar to cell division cycle protein cdc16 - Apc6	CMF102C
similar to chaperone grpE	CML088C
similar to DnaJ family molecular chaperone	CMB036C
similar to dnaJ protein	CMF076C
similar to general negative regulator of transcription cdc39	CMF162C
similar to heat shock protein Hsp33	CMR268C
similar to mitochondrial inner membrane protease IMP1	CMB077C
similar to nuclear import protein NPL4	CMH273C
similar to processing protease	CMO083C
similar to protease	CMO341C
similar to protein with DnaJ domain	CMI136C
similar to ribosomal protein S1	CMI199C
similar to ribosome biogenesis protein SSF1	CMQ177C
similar to ring finger protein	CME171C
similar to SUMO-1-specific protease	CML324C
similar to translation initiation factor IF-3	CMT223C
similar to translational activator GCN1	CMH194C
similar to ubiquitin carboxyl-terminal hydrolase 10	CMP333C
similar to ubiquitin conjugating enzyme E2	CMP178C
similar to ubiquitin conjugating enzyme E2	CMQ038C
similar to ubiquitin fusion-degradation protein 1	CMK008C

	small heat shock protein, hsp20 family	CMJ101C
	Smt3-conjugating enzyme E2	CMK193C
	ubiquitin carboxy-terminal hydrolase L3	CMQ026C
	ubiquitin carboxy-terminal hydrolase L5	CMO018C
	ubiquitin protein ligase E3A	CMB070C
	ubiquitin-activating enzyme E1	CMI034C
	ubiquitin-like protein Rub1	CML042C
	ubiquitin-specific protease	CMR057C
	ubiquitin-specific protease	CMR384C
	unknown zinc finger protein - SYVN	CMG190C
	UV-damaged DNA binding protein -DBB	CMR264C
Lipid-fatty acid metabolism	1-acylglycerol-3-phosphate O-acyltransferase	CMF185C
	3-hydroxybutyryl-CoA dehydrogenase	CMC137C
	3-hydroxybutyryl-CoA dehydrogenase	CMC137C
	3-oxoacyl-[acyl-carrier-protein] synthase I, precursor	CMM286C
	3-oxoacyl-[acyl-carrier-protein] synthase I, precursor	CMR500C
	acetyl-CoA acetyltransferase	CMA042C
	acetyl-CoA carboxylase beta subunit (accD)	CMV207C
	acetyl-CoA carboxylase carboxyltransferase alpha subunit (accA)	CMV056C
	acyl-CoA dehydrogenase, mitochondrial precursor	CML080C
	CDP-diacylglycerol--glycerol-3-phosphate 3-phosphatidyltransferase	CMN196C
	CDP-diacylglycerol--Inositol 3-phosphatidyltransferase	CMM125C
	CMN061Csimilar to 1-acylglycerol-3-phosphate O-acyltransferase - lysophospholipid acetyltrnsferase	CMP111C
	CMQ199Cdelta12 fatty acid desaturase	CMQ199C
	delta12 fatty acid desaturase	CMK291C
	enoyl-[acyl-carrier-protein] reductase [NADH]	CMT381C
	glycerol-3-phosphate O-acyltransferase	CMJ027C
	hypothetical protein - acyltransferase	CMB069C
	hypothetical protein - phosphatidic acid phosphatase	CMN061C
	long chain acyl-CoA synthetase	CME186C

	malonyl-CoA:Acyl carrier protein transacylase	CMT420C
	malonyl-CoA:Acyl carrier protein transacylase	CMT312C
	mitochondrial acyl carrier protein precursor, NADH-ubiquinone oxidoreductase 9.6 kD subunit	CMS372C
	mitochondrial ribosomal protein L15 precursor - acyl carrier protein (AcpP)	CMC132C
	phosphatidic acid phosphatase	CMR054C
	phosphatidyl-dlycerolphosphate synthase	CMJ134C
	phosphatidyl-glycerol phosphate phosphatase	CMJ134C
	phosphoethanolamine cytidylyltransferase	CMS052C
	probable choline kinase - ethanolamine kinase	CMR011C
	probable glycosyl transferase (ycf82)	CMV121C
	probable phosphatidate cytidylyltransferase - CDP-diacylglycerol synthase	CMM311C
	probable steroid dehydrogenase	CMK172C
	similar to 1-acylglycerol-3-phosphate O-acyltransferase - lysophospholipid acetyltrnsferase	CMR130C
	similar to 1-acylglycerol-3-phosphate O-acyltransferase - lysophospholipid acetyltrnsferase	CME109C
	similar to acyltransferase Tafazzin	CMP142C
	similar to nuclear receptor binding factor-like protein - trans-2-enoyl-CoA-reducatse	CMO278C
	similar to phosphatidic acid phosphatase	CMR488C
	sulfoquinovosyldiacylglycerol biosynthesis protein	CMR015C
	UDP-sulfoquinovose synthase, chloroplast precursor	CMR012C
Energetic metabolism	acetyl-CoA synthetase	CMK098C
	Aldehyde dehydrogenase	CMO345C
	aldehyde dehydrogenase 11A3	CMT034C
	Aldehyde reductase	CMM296C
	apcA	CMV158C
	apcB	CMV159C
	apcE	CMV157C

apcF	CMV048C
Apospory-associated protein C	CMT316C
atpA	CMV225C
atpD	CMV224C
atpF	CMV223C
atpG	CMV222C
atpH	CMV221C
cpcA	CMT009C
cpcB	CMV064C
cpcG	CMV051C
cytochrome b6/f complex iron-sulfur subunit precursor	CMO066C
cytochrome b6/f complex iron-sulfur subunit precursor	CMQ165C
cytochrome c oxidase assembly protein	CMS431C
Cytochrome c oxidase assembly protein COX11	CMG064C
Cytochrome c oxidase subunit VIa	CMJ036C
Cytochrome c oxidase subunit VIb	CMR404C
cytochrome c reductase cytochrome c1 subunit	CMP152C
cytochrome c reductase hinge protein	CMG215C
Cytochrome c reductase subunit 7(VI)	CMK031C
Cytochrome c reductase subunit 9(X)	CMD048C
cytochrome c reductase ubiquinone-binding protein	CMR178C
delta-aminolevulinic acid dehydratase	CMD104C
Dihydrolipoamide acetyltransferase component of pyruvate dehydrogenase complex	CMI273C
Dihydrolipoamide acetyltransferase component of pyruvate dehydrogenase complex	CMN017C
Dihydrolipoamide dehydrogenase	CMQ234C
Enolase	CMK131C
Ferrodoxin (petF)	CMV193C
Fructose-1,6-biphosphate aldolase	CME145C
Fructose-1,6-bisphosphatase	CMD041C
Fructose-bisphosphate aldolase	CMI049C

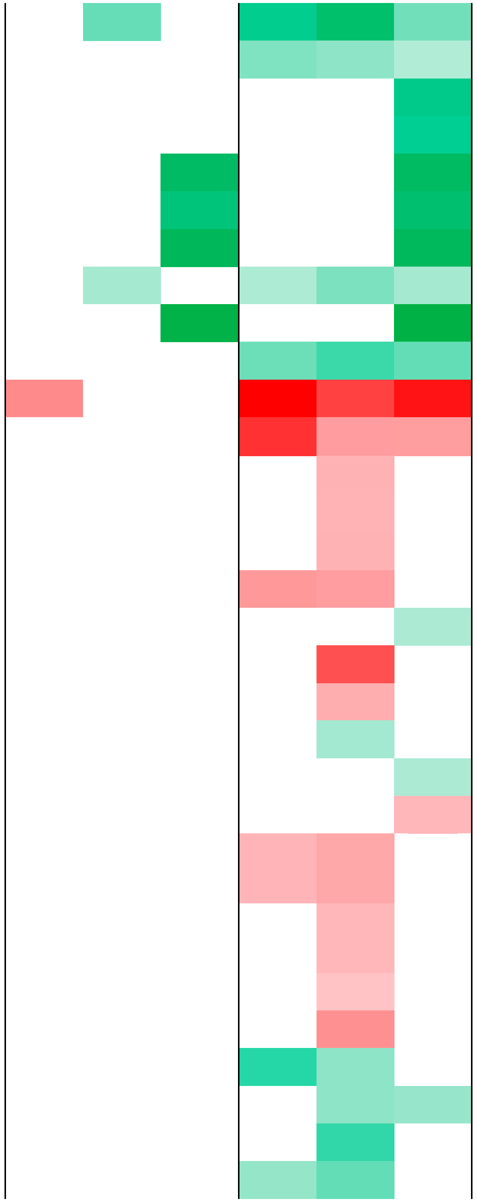

Glucose-6-phosphate isomerase	CMT497C
glutamyl-tRNA reductase	CMJ054C
Glyceraldehyde-3-phosphate dehydrogenase	CMM167C
L-lactate dehydrogenase	CMA145C
L-lactate dehydrogenase	CMJ002C
L-lactate dehydrogenase	CMK006C
Mg chelatase subunit H	CMB093C
Mg chelatase subunits I and D	CMM270C
Mg-protoporphyrin O-methyltransferase	CMI038C
Mg-protoporyphyrin IX chelatase (chlI)	CMV024C
mitochondrial F1FO ATP synthase subunit ATP5	CMG004C
mitochondrial F-type ATPase F1 subunit delta, precursor	CMN127C
mitochondrial F-type ATPase F1 subunit gamma, precursor	CML029C
NADH dehydrogenase I alpha subcomplex 2	CMI200C
NADH dehydrogenase I alpha subcomplex 8	CMA090C
NADH dehydrogenase I alpha subcomplex subunit 11	CMA128C
NADH dehydrogenase I beta subcomplex 7	CMC099C
NADH dehydrogenase I flavoprotein 24kDa subunit	CMR188C
NADH dehydrogenase I iron-sulfur protein 49kDa subunit	CMJ211C
NADH dehydrogenase type II	CMQ432C
NADH-ubiquinone oxidoreductase 9.6 kD subunit	CMS372C
oxygen independent coproporphyrinogen III oxidase	CMR445C
petA	CMV155C
petB	CMV096C
petD	CMV097C
petG	CMV139C
petL	CMV235C
petM	CMV147C
petN	CMV148C
PGR5	CMT289C
Phosphoenolpyruvate synthase	CMF012C
Phosphoglucomutase	CMJ272C

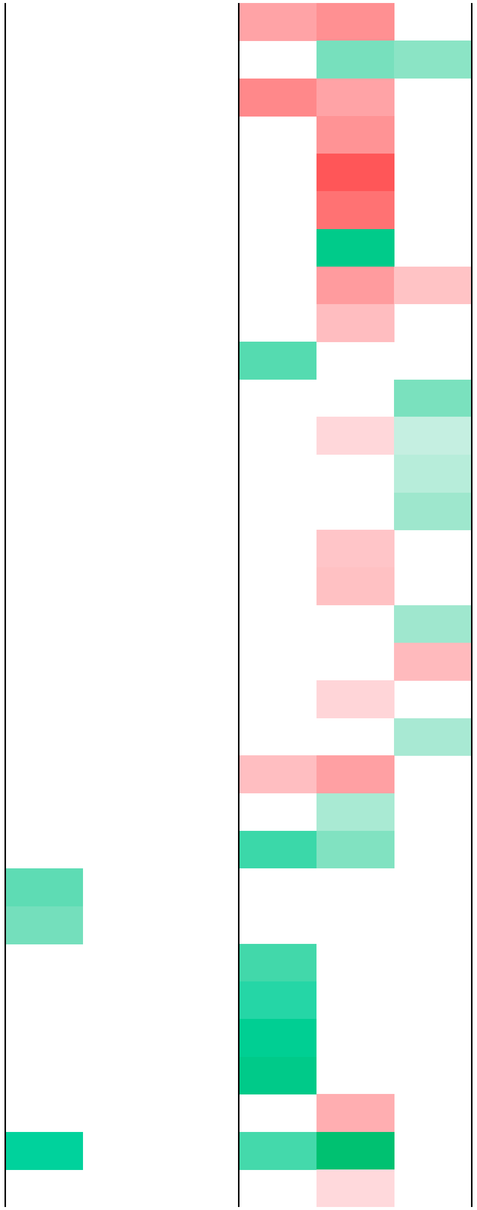

Phosphoglucomutase	CMT285C
Phosphoglycerate mutase	CMK188C
phycocyanin-associated rod linker protein	CMP166C
phycocyanobilin lyase alpha subunit	CMJ043C
phytoene desaturase	CMK151C
polyphosphate kinase	CMM026C
probable carotenoid cis-trans isomerase, CrtH	CMN268C
psaA	CMV135C
psaB	CMV136C
psaC	CMV059C
psaF	CMV201C
psaK	CMV055C
psaL	CMV236C
psaM	CMV028C
psba (D1)	CMV047C
psbB	CMV124C
psbC	CMV082C
psbD (D2)	CMV081C
psbE	CMV231C
psbF	CMV230C
psbH	CMV127C
psbI	CMV240C
psbJ	CMV228C
psbM	CMT182C
psbN	CMV126C
psbO	CMI290C
psbP	CMT368C
psbQ	CMC133C
psbT	CMV125C
psbU	CMI248C
psbV	CMV208C
psbX	CMV206C

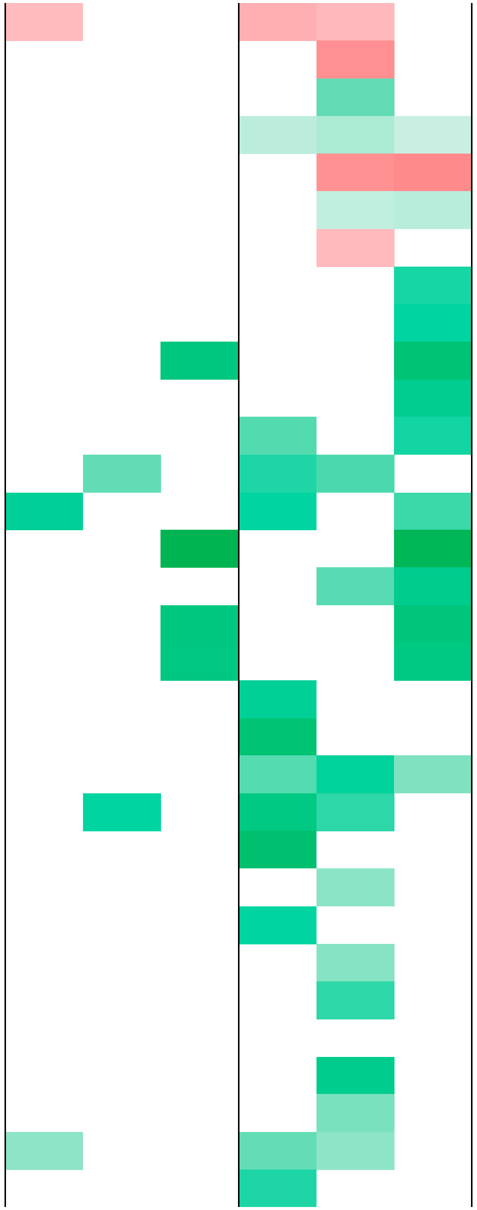

	psbZ	CMV141C
	Pyrophosphate--fructose 6-phosphate 1-phosphotransferase	CMH052C
	Pyrophosphate--fructose-6-phosphate 1-phosphotransferase	CMM196C
	Pyruvate dehydrogenase E1 component subunit alpha	CMT256C
	Pyruvate kinase	CMA030C
	Pyruvate kinase	CMK041C
	Pyruvate kinase	CMP260C
	Similar to cytochrome c	CMJ212C
	similar to V-type ATPase V1 subunit G	CMH200C
	succinate dehydrogenase flavoprotein subunit precursor	CMT582C
	Transketolase family protein	CMS327C
	V-type ATPase V0 subunit A	CMD095C
	V-type ATPase V0 subunit C	CMQ323C
	V-type ATPase V1 subunit B	CMP123C
	Ycf4	CMV092C
Metal transporter and complexing	ABC transporter, LktB-related	CMT120C
	ATP-binding cassette, sub-family A	CMH235C
	ATP-binding cassette, sub-family B - CmATM/HMT-1	CMN105C
	ATP-binding cassette, sub-family C, member 1 - CmMRP1	CMN251C
	ATP-binding cassette, sub-family D	CMP028C
	ATP-binding cassette, sub-family E, member 1	CMI058C
	ATP-binding cassette, sub-family G	CMD135C
	ATP-binding cassette, sub-family G	CMN295C
	ATP-binding subunit of unknown ABC transporter	CMO105C
	hypothetical protein - cmCOPT1	CMS307C
	probable manganese transporter - CmMTP2	CMC075C
	probable metal ion transporter - CmNRAMP2	CML262C
	probable zinc transporter - CmZIP1	CMS155C
	similar to cation transporting ATPase - P-type ATPase, subfamily V	CMR432C
	Similar to high-affinity iron permease - cmFTR2	CML004C
	similar to high-affinity iron permease - cmFTR4	CMN003C
	similar to iron transporter ferroportin 1 - cmIREG1	CMG212C

similar to metal ion transporter - CmNRAMP1	CMJ138C
Similar to periplasmic protein p19	CMG092C
similar to transmembrane calcium/manganese transporter Ccc1p	CMT466C
similar to zinc transporter - CmZIP2	CMG102C
sodium-dependent transporter	CML032C
sulfate binding protein of chloroplast sulfate ABC transporter	CMQ167C
unknown periplasmic-binding protein of ABC transporter	CMQ368C

Table S2. DEGs related to DNA maintenance. The table shows the total number of differentially expressed genes involved in DNA maintenance obtained from 1 and 3 mM Ni-treated cells at various time points. The DEGs are stratified across four categories: Repair, Replication, Recombination and Regulation. Repair and Replication categorizes entries with DNA repair or replication functions. The Recombination category groups all DEGs with a role in recombination at the DNA or chromosomal level. Regulation category encompasses DEGs with functions related to cell cycle, chromosome segregation, and histone modification. In cases where DEGs have multiple functions (e,g,, helicase), the entry is reported in all relevant categories. All entries report the gene name and locus (with CYME_ removed, for *C. merolae* genes). Where appropriate, the functional analysis of a gene is refined to either the functional domain of the protein or the protein itself. Tables S1 and S2 provide the basis for Figures 5 and 6 of the main manuscript, respectively.

	1 mM D5	1 mM D10	1 mM D15	3 mM D5	3 mM D10	3 mM D15
Upregulated	8	2	0	23	42	42
Downregulated	1	0	1	11	24	3

Upregulated DEGs							
1 mM Ni Day 5							
Repair		Replication		Recombination		Regulation	
CMA093C	ATP-dependent DNA helicase RecQ	CMO137C	probable DNA replication licensing factor	CMA093C	ATP-dependent DNA helicase RecQ	CMN029C	Sister chromatid cohesion protein Dcc1

			MCM2 - MCM9 like				
CMG028C	similar to meiotic nuclear division protein Mnd1	CMQ381C	DNA replication complex GINS protein Psf2				
		CMR406C	GINS complex subunit Sld5				
		CMT087C	similar to DNA replication licensing factor MCM8				
**1 mM Ni Day 10**							
**Repair**		**Replication**		**Recombination**		**Regulation**	
						CMN027C	probable chromosome segregation protein CSE1
						CMR026C	probable DNA cytosine-5 -methyltransferase 3
**3 mM Ni Day 5**							
**Repair**		**Replication**		**Recombination**		**Regulation**	
CMA093C	ATP-dependent DNA helicase RecQ	CMA093C	ATP-dependent DNA helicase RecQ	CMA093C	ATP-dependent DNA helicase RecQ	CMG143C	similar to anti-silencing factor - Histone chaperone ASF1-like
CMC115C	hypothetical protein – putative formation of a complex with endonuclease activity	CMF110C	replication factor C subunit 2	CMH155C	probable ATP-dependent DNA helicase RecQ	CMN027C	probable chromosome segregation protein CSE1
CMF110C	replication factor C subunit 2	CMH082C	DNA polymerase	CMI177C	hypothetical protein -	CMN029C	Sister chromatid cohesion protein Dcc1

			epsilon, subunit B		Structure-specific endonuclease subunit Slx4		
CMG028C	similar to meiotic nuclear division protein Mnd1	CMH155C	probable ATP-dependent DNA helicase RecQ	CMK235C	DNA ligase I	CMP244C	hypothetical protein - Mad1
CMG133C	excinuclease ABC subunit B (UvrABC system, subunit B)	CMI177C	hypothetical protein - Structure-specific endonuclease subunit Slx4			CMQ472C	similar to chromatin accessibility complex 1
CMH155C	probable ATP-dependent DNA helicase RecQ	CMK235C	DNA ligase I			CMR026C	probable DNA cytosine-5 -methyltransferase 3
CMI177C	hypothetical protein - Structure-specific endonuclease subunit Slx4	CMO137C	probable DNA replication licensing factor MCM2 - MCM9 like				
CMJ130C	probable cryptochrome DASH	CMQ381C	DNA replication complex GINS protein Psf2				
CMK235C	DNA ligase I	CMR136C	hypothetical protein - DNA replication complex GINS protein PSF1				
CMN306C	hypothetical protein - Fanconi	CMS124C	valosin-containing				

	anaemia protein FANCD		protein - AAA+ ATPase domain				
		CMT087C	similar to DNA replication licensing factor MCM8				
**3 mM Ni Day 10**							
**Repair**		**Replication**		**Recombination**		**Regulation**	
CMA093C	ATP-dependent DNA helicase RecQ	CMH155C	probable ATP-dependent DNA helicase RecQ	CMH155C	probable ATP-dependent DNA helicase RecQ	CMA089C	chromosome assembly complex Condensin II, subunit G2
CMC115C	hypothetical protein - to form a complex with endonuclease activity	CMK133C	hypothetical protein - DNA2/NAM7-like helicase	CMK133C	hypothetical protein - DNA2/NAM7-like helicase	CMA127C	cyclin-dependent kinase regulatory subunit
CMD139C	similar to endonuclease III	CMN138C	probable AAA protein spastin	CMS368C	DNA repair protein RAD52	CMB012C	similar to spindle pole body associated protein - Kinetochore-Ndc80 subunit Nuf2
CME067C	exonuclease I - endonuclease	CMN199C	DNA polymerase delta, catalytic subunit			CMB013C	topoisomerase II
CME176C	hypothetical protein - TFIIH subunit Tfb1/GTF2H1	CMO137C	probable DNA replication licensing factor MCM2 - MCM9 like			CMB064C	probable spindle checkpoint protein kinase MPS1
CMH155C	probable ATP-dependent DNA helicase RecQ	CMP212C	hypothetical protein - GINS complex, subunit Psf3			CMB108C	spindle checkpoint protein kinase MPS1

CMJ130C	probable cryptochrome DASH	CMQ381C	DNA replication complex GINS protein Psf2			CMD159C	similar to separin
CMK133C	hypothetical protein - DNA2/NAM7-like helicase	CMR136C	hypothetical protein - DNA replication complex GINS protein PSF1			CME029C	chromosome assembly complex Condensin, core subunit C - SMC4
CMN306C	hypothetical protein - Fanconi anaemia protein FANCD	CMT087C	similar to DNA replication licensing factor MCM8			CME082C	similar to cell division cycle protein CDC6
CMP065C	DNA excision repair protein ERCC2/XPD					CMG078C	hypothetical protein, conserved - Kinetochore protein Ndc80p
CMS368C	DNA repair protein RAD52					CMG186C	hypothetical protein - Kinetochore-Ndc80 subunit Spc24
CMT290C	similar to TFIIH subunit SSL1					CMG189C	chromosome assembly complex Condensin, core subunit E - SMC2
						CMH166C	DNA gyrase subunit B
						CMI099C	hypothetical protein - spindle and kinetochore-associated protein 1 SKA1
						CMI101C	hypothetical protein - Kinetochore protein Spc25
						CMI207C	chromosome assembly complex Condensin II, subunit H2
						CMI227C	probable G2/mitotic-specific cyclin 1

						CMK144C	hypothetical protein - Mitotic spindle checkpoint protein Bub1/Mad3
						CMP244C	hypothetical protein - Mad1
						CMQ236C	chromosome assembly complex Condensin II, subunit D3
						CMR026C	probable DNA cytosine-5 -methyltransferase 3
						CMS422C	chromosome assembly complex Condensin I, subunit G
**3 mM Ni Day 15**							
**Repair**		**Replication**		**Recombination**		**Regulation**	
CMA066C	DNA repair protein SMC6	CMA093C	ATP-dependent DNA helicase RecQ	CMA093C	ATP-dependent DNA helicase RecQ	CME082C	similar to cell division cycle protein CDC6
CMA093C	ATP-dependent DNA helicase RecQ	CMB052C	DNA polymerase delta, subunit B	CMC123C	probable replication protein A	CMG143C	similar to anti-silencing factor - Histone chaperone ASF1-like
CMB035C	mutS family DNA mismatch repair protein MSH4	CMC123C	probable replication protein A	CMH155C	probable ATP-dependent DNA helicase RecQ	CMI192C	sister-chromatide cohesion complex Cohesin, subunit SMC1
CMC123C	probable replication protein A	CMD025C	DNA replication licensing factor MCM2	CMI291C	similar to replication protein A 30kDa (RFA2-like)	CMK144C	hypothetical protein - Mitotic spindle checkpoint protein Bub1/Mad3

CMG028C	similar to meiotic nuclear division protein Mnd1	CMF127C	probable DNA polymerase alpha, subunit B			CML027C	sister-chromatide cohesion complex Cohesin, subunit SMC3
CMG106C	probable flap structure-specific endonuclease 1 - XPG/Rad2 endonuclease	CMF173C	DNA replication licensing factor MCM5			CML181C	hypothetical protein - E2F/DP family/ Transcription factor DP
CMG133C	excinuclease ABC subunit B (UvrABC system, subunit B)	CMH155C	probable ATP-dependent DNA helicase RecQ			CML190C	cell division cycle protein cdc23
CMH155C	probable ATP-dependent DNA helicase RecQ	CMI176C	DNA polymerase alpha, catalytic subunit			CMN029C	Sister chromatid cohesion protein Dcc1
CMH246C	probable DNA repair protein SPR18 - Structural maintenance of chromosomes protein 5 - SMC5	CMI291C	similar to replication protein A 30kDa (RFA2-like)			CMN029C	Sister chromatid cohesion protein Dcc1
CMI229C	mutS family DNA mismatch repair protein MSH6	CMJ261C	DNA replication licensing factor MCM6			CMP244C	hypothetical protein - Mad1
CMI291C	similar to replication protein A 30kDa (RFA2-like)	CMK284C	DNA primase, large subunit			CMQ139C	hypothetical protein, conserved - RPA-related protein RADX

CMK199C	mutS family DNA mismatch repair protein MSH4	CMN138C	probable AAA protein spastin			CMR026C	probable DNA cytosine-5 -methyltransferase 3
CMP167C	hypothetical protein - E3 SUMO-protein ligase Nse2 (Mms21)	CMN138C	probable AAA protein spastin			CMS101C	probable proliferating cell nuclear antigen (PCNA)
		CMO137C	probable DNA replication licensing factor MCM2 - MCM9 like			CMS239C	hypothetical protein - ARID DNA-binding domain
		CMO313C	DNA primase, small subunit				
		CMP356C	DNA replication licensing factor MCM4				
		CMQ381C	DNA replication complex GINS protein Psf2				
		CMR234C	DNA replication licensing factor MCM7				
		CMR406C	GINS complex subunit Sld5				
		CMS112C	similar to origin recognition complex subunit 1				
		CMS124C	valosin-containing protein - AAA+ ATPase domain				

		CMT087C	similar to DNA replication licensing factor MCM8				
		CMT300C	similar to origin recognition complex subunit 4				
**Downregulated DEGs**							
**1 mM Ni Day 5**							
**Repair**		**Replication**		**Recombination**		**Regulation**	
						CMT193C	hypothetical protein - GNAT domain
**3 mM Ni Day 5**							
**Repair**		**Replication**		**Recombination**		**Regulation**	
CMA062C	DNA polymerase iota, RAD30	CMA062C	DNA polymerase iota, RAD30			CMH166C	DNA gyrase subunit B
CMG142C	similar to DNA apurinic or apyrimidinic site lyase ARP	CMT350C	DNA polymerase delta, subunit D			CML102C	histone acetyltransferase HAC108/MYST
CMH170C	probable class II DNA photolyase					CMO046C	similar to PCAF histone acetylase complex subunit
CMT350C	DNA polymerase delta, subunit D					CMQ063C	hypothetical protein, conserved - SOS response associated peptidase (SRAP)
						CMQ367C	similar to nucleosome/chromatin assembly factor C

						CMS194C	similar to cell polarity protein alp13 - MRG
						CMT193C	hypothetical protein - GNAT domain
**3 mM Ni Day 10**							
**Repair**		**Replication**		**Recombination**		**Regulation**	
CMC111C	transcription-repair coupling factor	CMR028C	replication factor C subunit 1 (RFC1)			CME139C	similar to spindle checkpoint protein Xmad1 - Mad1
CMG142C	similar to DNA apurinic or apyrimidinic site lyase ARP	CMT350C	DNA polymerase delta, subunit D			CMI252C	DNA topoisomerase I
CMH170C	probable class II DNA photolyase	CMT462C	bacterial DNA polymerase I			CML102C	histone acetyltransferase HAC108/MYST
CMI169C	probable DNA mismatch repair protein MutL/Mlh/Pms-like					CMM055C	hypothetical protein - Protein DEK
CMK251C	probable DNA repair protein RadA					CMN118C	hypothetical protein - S-adenosyl-L-methionine-dependent methyltransferase ML2640-like
CMR028C	replication factor C subunit 1 (RFC1)					CMN180C	similar to methyl transferase
CMS282C	DNA repair protein REV1, deoxycytidyl transferase					CMN257C	similar to DNMT1 associated protein 1

CMT350C	DNA polymerase delta, subunit D					CMO046C	similar to PCAF histone acetylase complex subunit
						CMO165C	TATA-box binding protein-associated factor 9
						CMQ063C	hypothetical protein, conserved - SOS response associated peptidase (SRAP)
						CMQ358C	similar to candidate tumor suppressor - ING family
						CMR172C	hypothetical protein - Extended PHD (ePHD) domain
						CMS194C	similar to cell polarity protein alp13 - MRG
						CMT147C	similar to protein N-methyltransferase - SET domain superfamily
						CMT193C	hypothetical protein - GNAT domain
**3 mM Ni Day 15**							
**Repair**		**Replication**		**Recombination**		**Regulation**	
						CMN118C	hypothetical protein - S-adenosyl-L-methionine-dependent methyltransferase ML2640-like
						CMQ367C	similar to nucleosome/chromatin assembly factor C

						CMS194C	similar to cell polarity protein alp13 - MRG
